# A dual-module person-centered nursing self-care bundle for hypertension management: development and expert validation

**DOI:** 10.1186/s12912-026-05377-2

**Published:** 2026-09-26

**Authors:** Ni Putu Ayu Ratna Dewi, Nursalam Nursalam, Sriyono Sriyono, Israfil Israfil, Komang Ardidhana Nugraha Putra

**Affiliations:** 1 Faculty of Nursing, Airlangga University, Jl. Dr. Ir. H. Soekarno, Mulyorejo, Surabaya, 60115 Indonesia; 2 Faculty of Health, Institute of Technology and Health Bali, Denpasar, 80227 Indonesia

**Keywords:** Hypertension, Patient care bundles, Person-centered nursing, Primary health care, Self care

## Abstract

**Background:**

Sustained hypertension self-care is central to long-term blood pressure control, but it often remains suboptimal in primary care because patients may experience difficulties with daily monitoring, lifestyle modification, medication adherence, family support, and routine follow-up. Existing self-management materials commonly provide education or monitoring support, but fewer are designed to connect patients’ daily home-based self-care records with structured nurse-led review during routine primary care visits. This study aimed to develop and validate a dual-module person-centered nursing-based self-care bundle for hypertension management in primary care.

**Methods:**

This was a sequential intervention-development and expert-validation study with qualitative and quantitative components. Phase I examined the contextual relevance of four predefined self-care domains, conducted focus group discussions with patients, family caregivers, and healthcare providers, and integrated stakeholder and preliminary expert input into patient and nurse modules. Phase II involved seven experts who assessed content, presentation, language, graphic design, and suitability as health-promotion media. For content, I-CVI and S-CVI/Ave were interpreted as content-validity indices; the same proportional calculations summarized domain-specific expert agreement for the four non-content domains. Qualitative comments guided module refinement.

**Results:**

The patient module was structured as a self-care guide, daily record, checklist, and communication tool; the nurse module included guidance for education, record review, monitoring, individualized feedback, documentation, and follow-up. Content-domain S-CVI/Ave values were 0.89 for the patient module and 0.91 for the nurse module. Domain-average agreement for presentation, language, graphic design, and media suitability ranged from 0.93 to 1.00, while overall multidomain agreement was 0.94 and 0.95, respectively. Three items below the prespecified 0.86 threshold were revised. Experts confirmed that the requested changes had been addressed; no repeat ratings or post-revision CVI estimates were obtained.

**Conclusion:**

Expert validation indicated acceptable content validity and high expert agreement across the four non-content domains for the assessed module versions; these non-content ratings reflect expert judgment rather than target-user usability. Further studies should examine feasibility, implementation fidelity, patient usability, nurse adherence, and preliminary effects on self-care behavior and blood pressure control.

**Clinical trial number:**

Not applicable.

**Supplementary Information:**

The online version contains supplementary material available at https://doi.org/10.1186/s12912-026-05377-2.

## Background

Hypertension remains a major global health problem and requires long-term management beyond pharmacological treatment to prevent cardiovascular complications and improve quality of life [1, 2]. Effective control depends on sustained self-care, including home blood pressure monitoring, medication adherence, diet, physical activity, stress management, and routine healthcare follow-up [3]. However, maintaining these behaviors in daily life is often difficult, particularly in primary care settings where education, monitoring, and follow-up may be constrained by patient literacy, family roles, sociocultural practices, and service capacity. Many patients experience barriers such as inadequate health literacy, variable motivation, sociocultural beliefs, limited family support, and inconsistent professional guidance [4, 5]. Recent evidence also indicates that hypertension self-care is shaped by sociocultural context, family support, and healthcare-system factors [6, 7].

Although several hypertension self-management interventions have been developed, most focus on single behavioral components such as medication adherence, dietary modification, physical activity, or blood pressure monitoring. Such approaches are useful but may be insufficient when implemented in isolation, because hypertension self-care requires continuous education, family involvement, behavioral reinforcement, and coordination across home, family, and healthcare contexts [7]. Evidence suggests that integrated approaches combining patient-centered education, family engagement, and nurse-led support can strengthen adherence, sustain behavior change, and enhance continuity between home and primary care [8–10].

Person-Centered Nursing (PCN) provides a relevant framework for such integrated interventions by emphasizing individualized care, shared decision-making, therapeutic relationships, and supportive care environments [11]. In hypertension management, PCN is critical because self-care is shaped by patients’ daily routines, values, beliefs, family roles, perceived barriers, and interactions with healthcare providers [12]. Rather than positioning patients as passive recipients of education, a PCN approach encourages nurses to understand patients’ lived contexts, negotiate realistic goals, and support decisions that are meaningful and feasible for each patient. PCN-based interventions should therefore facilitate communication, support patient participation, guide behavior change, and enable nurses to monitor patient progress in a systematic but individualized manner [13, 14].

Despite the increasing application of PCN principles in chronic disease management, limited evidence is available on interventions that simultaneously guide patient self-care at home and provide nurses with practical operational support in primary care. Existing hypertension self-management materials commonly provide education or self-monitoring assistance, but fewer are designed to function at the same time as patient-held self-care records, nurse-reviewed communication tools, and operational guides for person-centered follow-up. This limits continuity between patients’ daily self-care practices at home and professional support delivered during routine primary care visits. As a result, patients’ actual self-care difficulties may remain invisible during consultations, while nurses may lack a structured basis for giving individualized feedback and planning subsequent care. A dual-module approach may provide a mechanism for making patient-reported self-care practices visible, discussable, and actionable during routine primary care encounters [10, 15].

Integrating patient and nurse roles through a dual-module PCN-based self-care bundle could address this gap by linking home-based self-care activities with nurse-led education, review, feedback, and follow-up planning. In this study, the term “bundle” refers to four coordinated and mutually reinforcing self-care support components: self-monitoring assistance, lifestyle-modification education, medication-regimen monitoring, and motivation for routine healthcare visits. These components are operationalized through linked patient and nurse modules. The term describes the intervention structure and does not imply established implementation fidelity or clinical effectiveness. To address this gap, this study aimed to develop and expert-validate a dual-module PCN-based self-care bundle for hypertension management in primary care. The bundle included a patient module, serving as a practical self-care guide and monitoring tool, and a nurse module, serving as an operational guide for providing person-centered education, supporting shared decision-making, reviewing self-care records, evaluating patient progress, and documenting follow-up during routine primary care visits. Specifically, the study aimed to: (1) examine the contextual relevance of four predefined self-care support domains and refine their operational content using empirical findings and stakeholder perspectives; (2) develop a patient module and a nurse module integrating self-monitoring assistance, lifestyle-modification education, medication-regimen monitoring, and motivation for routine healthcare visits; and (3) assess content validity, clarity, presentation, language, graphic design, and suitability as health-promotion media through expert validation.

## Methods

### Study design

This study employed a sequential intervention-development and expert-validation design with qualitative and quantitative components to develop and validate a dual-module bundle for hypertension management in primary care. The study was conducted from December 2025 to March 2026 and consisted of two sequential phases: module development and expert validation. Phase I focused on module development, while Phase II focused on expert validation. This design was selected to ensure that the modules were empirically grounded, contextually relevant, and culturally appropriate, and to prepare them for subsequent feasibility testing in primary care. Integration of qualitative and quantitative evidence was undertaken at three points. First, empirical findings and qualitative findings from the FGDs were used to examine the contextual relevance of the four predefined self-care domains and refine module content and practical features. Second, preliminary expert consultation was used to align module content with PCN principles and the intended primary care context. Third, quantitative I-CVI and S-CVI/Ave results were interpreted alongside qualitative expert comments to determine whether each component should be retained, revised, clarified, or strengthened.

### Phase I: Development of the dual-module PCN-based self-care bundle

The development phase was conducted through four structured steps: identification of strategic self-care issues, focus group discussions, expert consultation, and integration of findings into the patient and nurse modules. The final product of this phase consisted of two complementary modules. The patient module was developed for patients with hypertension and family caregivers and included practical information on hypertension self-care, blood pressure monitoring, lifestyle modification, medication adherence, routine healthcare visits, family support, warning signs, and self-care checklists. The nurse module was developed as a guide for nurses to deliver person-centered education, facilitate patient engagement, monitor self-care implementation, evaluate patient progress, and document follow-up during routine primary care visits. Both modules were designed as a dual-module bundle linking home-based self-care with nurse-led review and support in primary care.

#### Step 1: Identification of strategic self-care issues

The four candidate self-care support domains were specified a priori and were not generated by the preceding quantitative analysis. Their selection was informed by self-care theory, the PCN framework, international hypertension-management recommendations, and evidence on the principal behavioral and service-related requirements of long-term hypertension care [16–20]. The domains comprised self-monitoring assistance, lifestyle-modification education, medication-regimen monitoring, and motivation for routine healthcare visits. Together, they were intended to address continuous monitoring, non-pharmacological management, treatment adherence, and continuity with primary healthcare services.

Their contextual relevance was subsequently examined in an unpublished cross-sectional needs assessment conducted between September and November 2025 among 152 adults aged 40–59 years with hypertension recruited through multistage random sampling from four primary healthcare centers in Gianyar Regency, Bali. The assessment indicated that patient, family, sociocultural, and healthcare-service factors were associated with the combined candidate self-care support construct, which was also associated with self-management ability. These findings supported the retention and contextual refinement of the four predefined domains rather than their de novo identification. Their operational content was subsequently refined through FGDs and expert consultation. Only the findings necessary to explain intervention development are summarized in the present article. Detailed methods, statistical analyses, effect estimates, and extended interpretation of the quantitative needs assessment are reserved for a separate manuscript currently under peer review and are not reproduced here. The theoretical, empirical, and contextual rationale for retaining the four candidate self-care support domains is presented in Supplementary Table 9.

#### Step 2: Focus Group Discussions (FGDs)

Two stakeholder-specific FGDs were conducted to clarify the findings of the preceding quantitative needs assessment and refine the content and implementation features of the patient and nurse modules. Participants were purposively selected using predefined eligibility criteria, identified through the participating primary healthcare centers, and contacted directly by the principal researcher via WhatsApp. No invited participant declined participation or withdrew from the study. The first FGD, conducted on 21 December 2025, involved four patients with hypertension and four family caregivers. The second, conducted on 29 December 2025, involved five primary care nurses and three primary healthcare leaders. Patients and family caregivers participated separately from healthcare personnel to minimize the influence of professional hierarchy and facilitate open discussion.

Both FGDs were conducted through Zoom, lasted approximately 90 min, and used stakeholder-specific semi-structured interview guides developed for this study. The guides were not formally pilot-tested. NPARD, a female doctoral nursing student and the principal researcher, moderated both discussions. She had completed formal training in qualitative research methods and qualitative data analysis. Before consent, participants were informed of her role and the purpose of the study. She had no prior personal or professional relationship with the participants. No co-moderator was involved, and no persons other than the participants, moderator, and research assistant attended the discussions.

A registered nurse serving as the research assistant supported technical coordination, maintained field notes, and prepared verbatim transcripts from the audio-video recordings. NPARD checked the transcripts against the recordings before analysis. Coding and category development were conducted using the original Indonesian transcripts. Only quotations selected for publication were translated into English by NPARD and reviewed by the research team for meaning equivalence. Transcripts were not returned to participants, and no post-analysis participant checking was conducted. Instead, during each session, the moderator summarized key points and invited participants to clarify or correct the researchers’ interpretations. No repeat FGDs were conducted. The English interview guides are provided in Supplementary File 1, and participant characteristics are presented in Supplementary Table 10. The FGDs were intended to obtain focused stakeholder input for intervention development rather than to generate a comprehensive qualitative theory. Formal thematic saturation was not assessed, and no claim of saturation is made. Sample adequacy was considered in relation to the focused study aim, participant specificity, depth of discussion, and complementary perspectives obtained across stakeholder groups [21].

#### Qualitative data analysis

An inductive–deductive approach was used. NPARD and KANP independently coded the transcripts, compared and consolidated the codes, and grouped related codes into descriptive categories. Findings were compared across stakeholder groups and subsequently mapped to the four predefined self-care support domains. Findings extending beyond these domains, including language clarity, family roles, cultural considerations, recording needs, nurse workflow, and follow-up mechanisms, were retained to refine both modules. Coding disagreements were resolved through discussion and, when necessary, consultation with a senior co-author. Data were managed and coded manually without qualitative data-analysis software. The resulting evidence-to-module mapping is presented in Supplementary Tables 2 and 3. The qualitative component was reported with reference to the Consolidated Criteria for Reporting Qualitative Research, and the completed checklist is provided in Supplementary File 4 [22].

#### Step 3: Expert Consultation for Initial Module Refinement

Preliminary drafts of the patient and nurse modules were reviewed by three consultants purposively selected based on relevant postgraduate specialist or doctoral qualifications, at least five years of academic or clinical experience, and expertise in hypertension care, cardiovascular nursing, self-care management, PCN, or mental health nursing. The group comprised a clinical cardiovascular nurse and educator, a cardiovascular nursing academic, and a mental health nursing academic. Their professional roles, qualifications, areas of expertise, years of relevant experience, contributions, and relationships to the authors are presented in Supplementary Table 11.

Each of the three preliminary consultants was affiliated with a different institution, and none shared an institutional affiliation with the authors. None had a supervisory, line-management, authorship, decision-making, or other study-related role. They were not members of the research team and were not involved in participant recruitment, data collection, data analysis, or interpretation of the study findings. The consultation was qualitative and advisory, focusing on module structure, content, activities, monitoring tools, nurse guidance, and alignment with PCN principles. The preliminary consultants did not assign numerical ratings and were not included in the formal expert-validation panel. Their comments informed preliminary refinement before formal validation by a separate seven-member panel using the structured validation questionnaire.

#### Step 4: Integration and module drafting

Evidence from the preceding quantitative needs assessment, FGDs, and expert consultation was integrated into two complementary modules. The patient module comprised seven sections covering module use, hypertension, access to services, sociocultural considerations, family support, the four self-care domains, and feedback and follow-up. It included blood-pressure records, self-care checklists, and spaces for recording symptoms, barriers, questions, and agreed goals. The nurse module comprised eight sections covering hypertension, PCN principles, service access, cultural context, the nursing role, implementation of the four domains, monitoring, feedback, and follow-up. It provided counseling prompts, checklist-review procedures, longitudinal scoring, documentation forms, and guidance for individualized goal setting. Plain-language instructions, check-box formats, guided completion, and optional family assistance were incorporated for patients requiring additional support. Detailed module contents and representative excerpts are provided in Supplementary File 5.

The modules were designed for daily patient recording and review during routine monthly hypertension visits. Patients record self-care activities, medication use, blood-pressure readings when available, symptoms, barriers, and questions. During each visit, the nurse reviews these records, identifies patient-prioritized concerns, provides individualized education and feedback, agrees on a feasible goal, and documents the follow-up plan. The proposed orientation and pilot evaluation sequence are described in Supplementary File 5; their time requirements and integration into routine workflows remain to be assessed in subsequent feasibility testing.

PCN was operationalized through five person-centered nursing processes: working with patients’ beliefs and values, sharing decision-making, providing holistic care, having a sympathetic presence, and engaging authentically [11]. These processes guided the integration of patient-held records, family involvement, counseling prompts, patient-selected priorities, shared goal setting, individualized feedback, and documented follow-up across the four bundle domains **(**Table 1**).**

**Table 1:** Integration of FGD and expert consultation into the dual-module bundle

Source of Evidence	Key Inputs / Findings	Translation into Patient Module	Translation into Nurse Module	PCN principle addressed
Prior empirical findings	Four theory-informed candidate domains were specified a priori: self-monitoring assistance, lifestyle-modification education, medication-regimen monitoring, and motivation for routine healthcare visits. The preceding needs assessment supported their retention and contextual refinement.	Practical self-care guidance, daily monitoring checklists, medication reminders, lifestyle instructions, blood pressure records, and visit preparation notes.	Operational guidance for education delivery, monitoring indicators, evaluation checklists, follow-up documentation, and feedback planning.	Providing holistic care.
Patient and Family FGD	Patients and families needed simple, culturally appropriate guidance for daily hypertension care, including barriers to adherence, family involvement, and topics to discuss during routine control visits.	Simplified language, practical examples, family support tips, warning signs, daily self-care checklist, and spaces for recording questions, symptoms, and barriers.	Counseling prompts for assessing patient preferences, perceived barriers, family support, home routines, and culturally influenced self-care practices.	Working with patients’ beliefs and values; providing holistic care; having a sympathetic presence.
Healthcare Provider FGD	Nurses needed structured tools to educate, monitor, and evaluate patient self-care because routine education was not always supported by documentation or follow-up mechanisms.	Patient-held records to facilitate communication between patients, families, and nurses during routine visits.	Session objectives, education scripts, monitoring forms, evaluation points, documentation guidance, and follow-up planning tools.	Engaging authentically; providing holistic care.
Expert Consultation	Experts emphasized alignment with PCN principles, clarity of behavioral strategies, anticipated compatibility with routine primary-care workflows, and user-oriented module design.	Goal-setting forms, self-monitoring activities, reflection spaces, clearer instructions, and patient-friendly self-care records.	Shared decision-making prompts, individualized feedback mechanisms, checklist review guidance, and structured follow-up procedures.	Sharing decision-making; engaging authentically; working with patients’ beliefs and values.
Integration & Drafting	Evidence and stakeholder input were combined to develop two connected modules that link home-based self-care with nurse-led follow-up in primary care.	Patient module as a self-care guide, daily checklist, monitoring record, and communication tool for home use and routine visits.	Nurse module as an operational guide for education, checklist review, barrier identification, monitoring, evaluation, feedback, documentation, and follow-up planning.	Providing holistic care; sharing decision-making; engaging authentically.

Note: FGD, focus group discussion; PCN, Person-Centered Nursing

### Phase II: Validation of the dual-module PCN-based self-care bundle

Formal expert validation assessed content validity and expert-rated suitability of presentation, language, graphic design, and health-promotion media. Seven experts were purposively selected based on their relevant expertise in hypertension care, nursing, health promotion, self-care management, and primary healthcare. The sample size was considered adequate for content validation, as previous methodological guidance has recommended expert panels of approximately 5–10 members to evaluate the relevance and clarity of intervention content [23]. The multidisciplinary composition was intended to capture complementary perspectives on clinical accuracy, anticipated applicability to nursing practice, behavioral support, and educational-media suitability. The experts evaluated the patient and nurse modules separately using the structured validation questionnaire.

#### Expert panel

The expert panel consisted of seven professionals. Experts were eligible if they met at least two of the following criteria: academic or clinical expertise in hypertension care, cardiovascular or community nursing, self-care management, health promotion, patient education, behavioral intervention, or primary healthcare services; experience in research, clinical practice, teaching, or intervention development related to chronic disease management; and willingness to review both modules using the validation checklist. Individual characteristics of the seven formal validators, including professional background and qualifications, primary area of expertise, years of relevant experience, current clinical or academic role and setting, and relationship to the research team, are presented in Supplementary Table 12.

The composition of the panel was designed to reflect the multidimensional nature of the modules. Clinical experts assessed the accuracy and relevance of hypertension-related content. Nursing and primary care experts contributed perspectives on the modules’ anticipated fit with routine primary care services. Health promotion and educational media experts assessed readability, presentation, and suitability as learning materials. Behavioral or mental health nursing experts reviewed the extent to which the modules supported patient engagement, motivation, self-care behavior, family involvement, and continuity of care. This multidisciplinary composition was intended to reduce the risk of narrow expert judgment and broaden expert appraisal across domains relevant to the intended primary-care context.

#### Validation procedure

The expert validation questionnaire comprised five conceptually distinct domains: content, presentation, language, graphic design, and health-promotion media suitability. Content assessed alignment with standards, competency coverage, accuracy, supporting materials, and currency; presentation assessed organization and completeness; language assessed clarity, communicativeness, appropriateness, and logical flow; graphic design assessed format, cover, and internal layout; and media suitability assessed appropriateness, effectiveness, and creativity as a health-promotion resource. An English version of the questionnaire is provided as Supplementary File 2.

The checklist used a four-point scale: 4 = appropriate without revision, 3 = appropriate with minor revision, 2 = requires major revision, and 1 = not appropriate. For the purposes of this study, ratings of 3 or 4 were dichotomized as acceptable because both indicated that the criterion could be retained, although a rating of 3 required minor, non-substantive refinement. Ratings of 1 or 2 were classified as unacceptable. This dichotomization was a prespecified, study-specific analytic rule and was not intended to represent a universal cut-point for presentation, language, graphic design, or health-promotion media suitability. With seven experts, agreement by at least six experts yielded an item-level agreement value of 0.86, which was used as the prespecified threshold. To preserve the distinction between endorsement without revision and acceptance requiring minor refinement, separate item-level counts of ratings of 3 and 4 were retained in addition to the dichotomized agreement values.

For the content domain, item-level and domain-average agreement were interpreted as I-CVI and S-CVI/Ave, respectively [23–26]. For presentation, language, graphic design, and health-promotion media suitability, the same calculations represented domain-specific expert agreement rather than content validity. The mean across all 20 items was reported descriptively as overall multidomain expert agreement and was not interpreted as a single content-validity estimate. Item-specific qualitative comments were collected alongside the expert rating for each validation criterion. The comments were compiled separately for the patient and nurse modules and reviewed together with the corresponding item-level ratings. For each item below the prespecified agreement threshold, the research team identified the specific expert concern, determined the corresponding revision, and documented the location of the change in the revised module.

Following revision, the updated modules were returned electronically to all seven experts for a qualitative post-revision check. The criterion for this check was whether each previously requested item-specific revision had been adequately addressed in the revised module. No repeat numerical ratings were requested or obtained; therefore, the post-revision check did not generate new I-CVI or S-CVI/Ave estimates and was not interpreted as repeat quantitative validation. The revised module versions were subsequently prepared for target-user usability and feasibility testing. The detailed refinements and post-revision structure of the patient and nurse modules are presented in the Results section.

### Ethical considerations

Ethical approval for the overall research project was obtained from the Ethics Committee of the Institute of Technology and Health Bali (No. 03.322/KEPITEKES-BALI/IX/2025). The same ethical approval covered both the preceding quantitative needs assessment involving 152 adults with hypertension and the subsequent module-development and expert-validation procedures reported in the present study. All participants were informed about the objectives and procedures relevant to their participation, the voluntary nature of participation, and data confidentiality. Written informed consent was obtained from participants before data collection in each component of the research. The research was conducted in accordance with the principles of the Declaration of Helsinki and applicable ethical guidelines.

## Results

### Phase I: Development of the dual-module PCN-based self-care bundle

The development phase resulted in a dual-module bundle comprising a patient module and a nurse module. The process followed four sequential steps: identification of strategic self-care issues, FGDs with stakeholders, expert consultation for initial refinement, and integration of findings into the module drafts.

The identification of strategic issues revealed several factors that contributed to suboptimal hypertension self-management, including limited patient knowledge and self-efficacy, inadequate family support, sociocultural beliefs and habits, constrained healthcare service support, and inconsistent implementation of self-care practices. These findings indicated the need for an intervention that addressed patients’ daily self-care practices at home and supported nurses in reviewing, reinforcing, and following up those practices during routine primary care visits. Four candidate bundle domains were retained as core components: self-monitoring assistance, lifestyle-modification education, medication-regimen monitoring, and motivation for routine healthcare visits. These domains aligned with PCN principles, including working with patients’ beliefs and values, sharing decision-making, providing holistic care, having a sympathetic presence, and engaging authentically.

FGDs confirmed that hypertension self-care practices were fragmented and often concentrated on medication-taking rather than comprehensive daily self-management. Patients reported that blood pressure monitoring, diet control, physical activity, stress management, and routine follow-up were not consistently practiced. One patient stated, “I usually take my medicine, but I do not regularly record my blood pressure unless I feel unwell” (Patient 2). Another patient described the difficulty of maintaining lifestyle changes during social and cultural events: “It is difficult to control food during family or traditional events because I do not want to refuse what is served” (Patient 3). Family caregivers also reported that their support was often limited to reminding patients to take medication or accompanying them to healthcare visits. As one caregiver explained, “We mostly remind the patient to take medicine, but we are not sure how to help with diet or exercise” (Family caregiver 1). Healthcare providers highlighted that hypertension education was usually delivered during routine visits, but structured tools for monitoring patients’ home-based self-care were limited. A nurse stated, “We give education during visits, but we do not have a specific record to see what patients actually do at home every day” (Nurse 2). A primary healthcare coordinator also noted, “Follow-up is sometimes difficult because of workload and the absence of a practical tool that connects patient activities at home with our evaluation in the clinic” (Healthcare coordinator 1). These stakeholder perspectives showed that patients needed a simple way to document daily self-care, while nurses needed a practical basis for reviewing patient-reported barriers and providing individualized feedback during routine visits.

These findings informed the development of two complementary modules. The patient module was designed as a patient-held self-care guide, record, and communication tool for daily home use and review during routine monthly visits. It included the four self-care domains, blood-pressure and self-care records, and spaces for documenting symptoms, barriers, questions, family-support needs, and agreed follow-up. The nurse module provided structured guidance for education, record review, barrier identification, monitoring, individualized feedback, documentation, and follow-up planning. Together, the modules were designed to link patients’ home-based self-care records with nurse-led support during routine primary care visits. Their structure and operational use are summarized in Table 2, with detailed evidence-to-module mapping provided in Supplementary Tables 1–3.

**Table 2:** Structure and function of the dual-module bundle

Component	Patient module	Nurse module
Main purpose	To guide patients and families in implementing hypertension self-care at home and recording self-care progress.	To guide nurses in providing PCN-based education, monitoring, evaluation, feedback, and follow-up.
Target users	Patients with hypertension and family caregivers.	Nurses and healthcare providers in primary care.
Main function	Educational guide, self-care record, communication tool, and monitoring booklet.	Operational guide for education delivery, checklist review, barrier identification, documentation, and follow-up planning.
Core content	Hypertension education, PCN-based communication, service access, cultural context, family support, bundle implementation, checklist, and follow-up plan.	Hypertension education, PCN principles, counseling guidance, monitoring indicators, evaluation checklist, documentation form, and follow-up guidance.
Bundle domains	Self-monitoring assistance; lifestyle-modification education; medication-regimen monitoring; motivation for routine healthcare visits.	Self-monitoring assistance; lifestyle-modification education; medication-regimen monitoring; motivation for routine healthcare visits.
Use during routine visits	Patients bring completed records and checklists for review and discussion.	Nurses review records, identify difficulties, discuss patient-selected topics, provide feedback, and document follow-up plans.
Frequency of use	Daily home recording and monthly clinic discussion.	Monthly review during routine primary care visits.
Expected output	Completed checklist, BP record, questions, and barriers.	Feedback, follow-up plan, and documentation.
Proposed pilot sequence	Daily home recording over three months, with monthly review	Baseline orientation followed by reviews at months 1, 2, and 3
Intended routine use	Continued daily recording if subsequent testing supports implementation	Review during routine monthly hypertension visits

Note: BP, blood pressure; PCN, Person-Centered Nursing

The five PCN processes were translated into specific module components and reciprocal patient–nurse activities rather than being used only as general theoretical principles. The relationship between PCN constructs, module components, patient and family activities, nurse activities, and intended proximal outcomes is presented in Table 3.

**Table 3:** Operationalization of the five PCN processes within the dual-module bundle

PCN process	Module component	Patient and family activity	Nurse activity	Intended proximal outcome
Working with patients’ beliefs and values	Sociocultural and family-support sections; fields for recording preferences, beliefs, barriers, questions, and support needs across the four self-care domains	Patients record daily routines, treatment beliefs, cultural practices, perceived barriers, and preferred forms of family support. Family members contribute according to the patient’s preference.	Explore the patient’s beliefs, values, treatment concerns, daily routines, cultural obligations, and preferred family involvement; adapt self-monitoring, lifestyle, medication, and visit-related recommendations to the patient’s context.	Greater contextual relevance and alignment of the self-care plan with the patient’s values and daily life.
Sharing decision-making	Patient-selected discussion priority; goal-setting form; individualized feedback and follow-up plan.	Patients identify the issue they wish to discuss first, select a feasible self-care priority, and participate in determining the preferred family-support role.	Present feasible options, discuss expected benefits and barriers, agree with the patient on a realistic goal, and document the agreed action and follow-up plan.	Increased patient participation, ownership, and agreement with the selected self-care goal.
Providing holistic care	Integrated records covering the four bundle domains, symptoms, stress, family support, sociocultural factors, access barriers, and follow-up needs.	Patients record blood-pressure monitoring, medication use, lifestyle practices, symptoms, emotional or practical difficulties, service-access barriers, and support needs.	Review clinical, behavioral, psychosocial, family, cultural, and service-related information together; identify priorities and arrange additional support or referral when required.	More comprehensive identification of self-care needs and a coordinated, individualized follow-up plan.
Having a sympathetic presence	Reflection spaces; fields for barriers, symptoms, concerns, and questions; person-centered counseling prompts.	Patients describe difficulties, concerns, symptoms, and experiences that may not be captured through fixed checklist responses.	Use open and non-judgmental questions, listen attentively, acknowledge the patient’s difficulties, clarify understanding, and respond without blame or coercion.	Greater trust, psychological safety, and disclosure of self-care difficulties.
Engaging authentically	Daily patient-held records; monthly record-based dialogue; individualized feedback, documentation, and continuing follow-up.	Patients complete the self-care records, bring them to routine visits, discuss progress and difficulties, and participate in reviewing the follow-up plan.	Review the patient’s actual records, discuss progress and barriers transparently, provide individualized feedback, confirm mutual understanding, and document subsequent actions.	Stronger therapeutic engagement, actionable patient–nurse communication, and continuity of self-care support.

Note: PCN, Person-Centered Nursing. The four bundle domains comprised self-monitoring assistance, lifestyle-modification education, medication-regimen monitoring, and motivation for routine healthcare visits. The intended proximal outcomes represent theory-informed mechanisms and were not measured in the present development and expert-validation study

### Phase II: Expert Validation of the dual-module PCN-based self-care bundle

Formal expert validation of the patient and nurse module versions before revision was conducted by seven experts with backgrounds in hypertension care, cardiovascular nursing, community or primary care nursing, self-care intervention, health-promotion media, behavioral nursing, and clinical practice. The validation covered five aspects: content, presentation, language, graphic design, and suitability as health-promotion media. The two modules were reviewed separately because the intervention was designed as an integrated package with two connected functions. The patient module was reviewed as a self-care guide, monitoring record, and communication tool, whereas the nurse module was reviewed as an operational guide for person-centered education, checklist review, monitoring, evaluation, individualized feedback, documentation, and follow-up planning.

The content domain demonstrated generally acceptable content validity, with S-CVI/Ave values of 0.89 for the patient module and 0.91 for the nurse module. Domain-average expert agreement for presentation, language, graphic design, and health-promotion media suitability ranged from 0.93 to 1.00. These non-content agreement values represent expert judgments of the assessed module versions and should not be interpreted as evidence of target-user usability or acceptability. Overall multidomain agreement across all 20 items was 0.94 for the patient module and 0.95 for the nurse module. These descriptive averages were not interpreted as measures of overall validity, usability, or clinical effectiveness; interpretation focused primarily on the domain-specific results. Most items met the prespecified item-level agreement threshold of ≥ 0.86. Three items had an agreement value of 0.71: alignment with standards and completeness of presentation in the patient module, and supporting learning materials in the nurse module.

For the patient module, experts requested clearer alignment between the content and the four predefined self-care domains and a more complete presentation of practical, recording, and follow-up elements. Accordingly, the relationship with the four domains was made more explicit, while the self-care checklists, blood-pressure and monitoring records, goal-setting spaces, and sections for symptoms, barriers, questions, and follow-up were strengthened. For the nurse module, experts requested more operational supporting materials; counseling prompts, checklist-review procedures, monitoring indicators, documentation forms, and follow-up guidance were expanded. Following revision, the modules were returned to all seven experts for a qualitative post-revision check to determine whether their requested changes had been addressed. All seven experts confirmed that the requested revisions had been addressed. The item-level audit trail linking each low-agreement item to the corresponding expert concern, specific revision, location in the revised module, and post-revision outcome is presented in Supplementary Table 8. No repeat numerical ratings were obtained. Accordingly, the reported content-validity and domain-specific agreement values apply only to the module versions assessed before revision, and no post-revision CVI estimates are available. Detailed item-level results, including separate counts of ratings of 3 and 4, are presented in Supplementary Tables 4 and 5 with domain-level results summarized in Table 4.

**Table 4:** Content validity and domain-specific expert agreement before module revision (*n* = 7)

Validation aspect	Number of items	Patient module		Nurse module		Interpretation
		Item-level agreement range	Domain-average agreement	Item-level agreement range	Domain-average agreement	
Content	5	0.71–1.00	0.89	0.71–1.00	0.91	Acceptable domain-average content validity; selected items required revision
Presentation	4	0.71–1.00	0.93	0.86–1.00	0.93	Presentation suitable with one patient-module item required revision
Language	6	0.86–1.00	0.95	0.86–1.00	0.98	Language suitable
Graphic design	3	0.86–1.00	0.95	1.00–1.00	1.00	Graphic-design suitable
Health- promotion media	2	1.00–1.00	1.00	0.86–1.00	0.93	Suitable as health-promotion media
Overall	20	0.71–1.00	0.94	0.71–1.00	0.95	Descriptive overall multidomain agreement; selected items required revision

Note: All reported I-CVI, S-CVI/Ave, and domain-specific agreement values refer to the module versions assessed before revision; no post-revision numerical ratings were obtained. Overall multidomain averages are descriptive only and should not be interpreted as measures of overall validity, usability, or clinical effectiveness; interpretation should prioritize the domain-specific results

## Discussion

The main contribution of this study is the operationalization of PCN through two linked tools for hypertension self-management: a patient-held module for daily self-care recording and a nurse-facing module for review, education, monitoring, individualized feedback, documentation, and follow-up. The patient module was designed to support self-monitoring, family involvement, and documentation of daily practices, barriers, symptoms, questions, priorities, and agreed goals. The nurse module was designed to provide a structured basis for reviewing these records, exploring patients’ beliefs and preferences, identifying barriers, adapting recommendations to individual contexts, and documenting agreed follow-up actions. Unlike a conventional education booklet plus checklist, the bundle operationalizes the five PCN processes through reciprocal patient–nurse activities rather than unidirectional information delivery. This structure was intended to address a gap in hypertension self-management support by linking patients’ home-based records with structured nurse review. Together, the modules were designed to support therapeutic engagement, shared decision-making, individualized care, and continuity between home-based self-care and primary care review.

The bundle was developed based on empirical findings, stakeholder input, expert consultation, and PCN principles. This multistep development process strengthened the contextual grounding of the intervention because the module content was derived from problems identified by patients, families, and healthcare providers, rather than from theoretical assumptions alone. Strategic issues and FGD findings showed that hypertension self-care was influenced by patient knowledge and self-efficacy, family support, sociocultural practices, healthcare service constraints, and the need for more consistent professional support. These findings are consistent with contemporary evidence showing that self-care is shaped by illness perception, family and social support, cultural context, access to professional guidance, and available resources, rather than solely by individual motivation [6, 27, 28]. By incorporating these factors into module content, checklists, recording spaces, and nurse review procedures, the development process translated PCN principles into structured module components intended for use in primary care.

A distinctive feature of the patient module is that it functions not only as an educational booklet but also as a patient-held self-care record and communication tool. It was designed to enable patients to document blood pressure monitoring, daily self-care activities, barriers, symptoms, questions, and follow-up needs for discussion with nurses during monthly routine visits [29]. This design is aligned with evidence that structured self-management support, monitoring, and feedback can strengthen patients’ confidence, engagement, and participation in hypertension care [5, 8]. The patient module was designed to provide a mechanism through which patients can bring their documented self-care experiences into consultations, including difficulties that might otherwise remain unreported [29]. This is important because routine clinical encounters may focus mainly on blood pressure values or medication use, while less visible issues such as dietary challenges, stress, family routines, cultural practices, and uncertainty about self-monitoring may receive limited attention. Therefore, the patient-held module may help improve patient–nurse communication by giving both parties a concrete record to review and discuss.

The nurse module was designed to strengthen the intervention by structuring the nurse’s role in hypertension self-care support. This emphasis is consistent with evidence that nurse-led interventions in primary care can improve hypertension management when nurses provide education, monitoring, behavioral support, and follow-up [10, 30, 31]. In the present FGDs, healthcare providers reported that routine hypertension education was not consistently supported by structured monitoring, individualized feedback, or follow-up documentation. The nurse module was developed to address this limitation by guiding nurses to review patient-completed checklists, identify self-care barriers, provide individualized feedback, document progress, and develop follow-up plans. This nurse-facing component is particularly important because patient-held tools may have limited value if healthcare providers do not actively review and respond to the information recorded by patients. The nurse module is therefore intended to operationalize PCN by translating therapeutic engagement, shared decision-making, and individualized care into structured steps for routine primary care encounters [11, 17]. This positions nurses not only as educators, but also as facilitators of self-care interpretation, behavioral reinforcement, and continuity of care.

Family involvement was an important component of the bundle [32, 33]. FGD findings revealed that family support often focused on accompanying patients to visits or reminding them to take medication, with less involvement in dietary regulation, physical activity, emotional adjustment, and long-term monitoring. Sociocultural practices also influenced daily routines, food choices, medication beliefs, and adherence behaviors [6, 34]. Accordingly, the patient module included family support guidance and culturally sensitive self-care strategies, while the nurse module included prompts for assessing family involvement and negotiating realistic lifestyle modifications. This approach aligns with evidence that family involvement can improve blood pressure management and adherence to lifestyle recommendations [7, 9]. For patients with hypertension, family routines and cultural obligations may either support or hinder self-care; therefore, interventions that ignore these contextual factors may be less feasible in daily life.

For the content domain, I-CVI and S-CVI/Ave quantified expert agreement regarding content relevance and representativeness [24, 26]. For the four non-content domains, the corresponding agreement values reflected presentation, language, graphic design, and media suitability rather than content validity. These non-content agreement values reflect expert appraisal only and should not be interpreted as evidence of target-user usability or acceptability. Formal expert validation demonstrated generally acceptable content validity for the content domain, with S-CVI/Ave values of 0.89 for the patient module and 0.91 for the nurse module. Expert agreement was also high for presentation, language, graphic design, and health-promotion media suitability, with domain-average values ranging from 0.93 to 1.00. However, two patient-module items and one nurse-module item fell below the prespecified item-level agreement threshold and were revised in response to item-specific expert comments. Methodological guidance describes content-validity indices as estimates derived from structured expert ratings [23–26]. Because the revised items were not quantitatively re-rated, no post-revision CVI estimates or quantitative evidence of improved content validity are claimed. The post-revision review had a narrower purpose: to determine whether the experts’ previously requested item-specific revisions had been addressed. All seven experts indicated that the requested revisions had been addressed; however, this finding represents a qualitative procedural check and should not be interpreted as evidence of post-revision validation. The revised module versions therefore require subsequent target-user usability and feasibility testing.

Qualitative feedback from experts was critical in refining both modules. Expert comments informed refinements to the operational specificity of the patient module, including revisions to self-care tasks, technical terminology, self-monitoring records, medication-related instructions, and spaces for documenting barriers, questions, symptoms, and follow-up needs. The nurse module was refined to clarify PCN-based counseling, review of patient-completed checklists, evaluation indicators, documentation, and follow-up planning. These revisions addressed expert-identified concerns related to clarity and suitability, although target-user usability remains to be evaluated. The revised bundle was theoretically grounded in PCN and prepared for subsequent usability and feasibility testing in routine hypertension care. However, its effects on patient engagement, adherence, self-management behavior, blood pressure control, and continuity of care remain to be evaluated in future feasibility and effectiveness studies.

## Implications

The findings have important implications for nursing practice and primary care services. The dual-module bundle was designed to provide a structured approach to linking patients’ daily home-based self-care records with nurse-led education, monitoring, feedback, and follow-up during routine visits. The patient-held self-care booklet may help patients and families document blood pressure monitoring, medication adherence, lifestyle practices, barriers, symptoms, questions, and topics for discussion. At the same time, the nurse module is intended to support individualized and person-centered guidance based on patients’ own records. For nurses in primary care, the bundle was designed as a structured resource to support record review, dialogue, individualized feedback, and follow-up planning within hypertension care. For primary care services, the nurse module may support more consistent hypertension self-care support by standardizing checklist review, barrier identification, documentation, and follow-up. This structure may be particularly relevant in settings where routine hypertension education is available but is not consistently supported by tools linking home-based self-care with clinical review. The proposed implementation includes nurse preparation regarding the five PCN processes, patient-record review, individualized feedback, and follow-up documentation. The planned initial patient orientation is approximately 10–15 min; however, actual time requirements for orientation and monthly record review, documentation burden, and compatibility with existing primary-care documentation systems remain to be established during feasibility testing.

## Strengths and limitations

This study has several strengths. First, the development process was systematic and sequential, integrating empirical findings, FGD data, expert consultation, and formal expert validation. Second, the intervention produced two complementary modules that explicitly linked patients’ daily home-based self-care records with nurse-led review, feedback, documentation, and follow-up in primary care. Third, the modules addressed not only clinical self-care behaviors but also communication, family involvement, cultural context, monitoring, and documentation, which may enhance their contextual relevance to primary care.

Several limitations should be considered. First, the qualitative component involved two stakeholder-specific FGDs with eight participants in each group, purposively recruited from a single regional setting. Although these participants provided focused perspectives from patients, families, nurses, and primary healthcare leaders, the findings may not represent broader populations or other healthcare contexts. Second, because participants discussed their experiences and expectations with the research team, social desirability bias cannot be excluded. Third, expert assessment may have been influenced by expert-consensus bias, and therefore the reported agreement indices should be interpreted as reflecting expert judgment rather than evidence of effectiveness or usability. Fourth, although two patient-module items and one nurse-module item with agreement values below the prespecified threshold were revised based on expert feedback, the revised items were not quantitatively re-rated; therefore, no post-revision CVI estimates or quantitative evidence of improvement are available. Fifth, direct target-user usability testing, including assessment of patient comprehension, acceptability, and practical use of the modules, was not conducted in this development phase. Finally, the feasibility of integrating monthly record review, documentation, and individualized feedback into routine primary-care workflows, including potential nurse workload implications, remains to be evaluated in subsequent feasibility studies.

## Conclusion

This study developed a dual-module PCN-based self-care bundle and conducted formal expert validation of the patient and nurse module versions. The bundle comprises a patient-held module for daily self-care recording and a nurse-facing module for person-centered education, record review, individualized feedback, documentation, and follow-up planning. Formal expert validation demonstrated generally acceptable content validity for the module versions assessed before revision and informed subsequent refinement. Expert agreement across the non-content domains reflected expert appraisal of presentation, language, graphic design, and media suitability and does not establish target-user usability or acceptability. After revision, the experts confirmed that their requested changes had been addressed; no repeat numerical ratings were obtained, and no post-revision CVI estimates were calculated. By linking patients’ home-based self-care records with nurse-led review during routine primary care visits, the bundle may provide a structured mechanism for supporting continuity of hypertension self-management. Further studies should examine target-user usability, feasibility, implementation fidelity, preliminary effectiveness, and sustainability before wider implementation.

## Supplementary Information

Below is the link to the electronic supplementary material.

Supplementary Material 1

Supplementary Material 2

Supplementary Material 3

Supplementary Material 4

Supplementary Material 5

## Data Availability

The de-identified datasets generated during this study, including focus group discussion transcripts, expert validation ratings, and module development materials, are available from the corresponding author upon reasonable request. The English versions of the semi-structured interview guide and expert validation questionnaire are provided as Supplementary Files 1 and 2, respectively. Supplementary File 3 contains Supplementary Tables 1–12, which provide detailed information on the development process, FGD findings, expert feedback, validation results, participant characteristics, and characteristics of the preliminary consultants and formal expert-validation panel. Supplementary File 4 contains the completed COREQ checklist. Supplementary File 5 provides English-language tables of contents and representative excerpts from both modules, including sample patient records, nurse review forms, counseling prompts, and the proposed review workflow. The complete patient and nurse modules were developed in Bahasa Indonesia. They are available from the corresponding author upon reasonable request, subject to copyright and institutional requirements.

## Abbreviations

COREQ: Consolidated Criteria for Reporting Qualitative Research
FGD: Focus group discussion
I-CVI: Item-level Content Validity Index
PCN: Person-Centered Nursing
S-CVI/Ave: Scale-level Content Validity Index/Average
