# Supplementary material for: A dual-module person-centered nursing self-care bundle for hypertension management: development and expert validation
Source: BMC Nurs. 2026 Sep 26;25:987. doi: 10.1186/s12912-026-05377-2 (PMC13673632; doi:10.1186/s12912-026-05377-2)
Supplement: Supplementary file 1 [file 12912_2026_5377_MOESM1_ESM.docx]

**Supplementary File 1**. Semi-Structured Interview Guide (English Version)

**FGD 1: Patients and Family Caregivers**

Participant Information:

Name: ___________________________

Role: Patient / Family Caregiver

Institution / Organization: ___________________________

Instructions:

This guide was used flexibly during the FGD. The main questions were used to guide discussion, while the probes were optional and used only when further clarification or elaboration was needed. The order of questions could be adjusted according to the flow of discussion. Before questions concerning the proposed bundle, the moderator provided a brief neutral overview of the four candidate domains without presenting preferred responses.

**Questions:**

**Patient Factors: Knowledge and Self-Efficacy**

*Core Question:*

A1. How do patients perceive the importance of self-care in managing hypertension?

*Optional probes:*

a. What daily actions do patients perform to manage hypertension?
b. Do patients feel they understand their condition well enough?
c. How do patients perceive the long-term impact of hypertension self-care on their health?
d. What role does the family play in reminding or motivating patients to perform self-care?

*Core Question:*

A2. What helps patients feel more confident in managing hypertension?

*Optional probes:*

a. What factors increase patients’ confidence in performing self-care?

b. How does family support contribute to patient adherence?

c. What challenges reduce patients’ confidence in managing hypertension?

**Family Factors: Coping, Support, and Role**

*Core Question:*

B1. How does family support influence patient self-care?

*Optional probes:*

a. Are family members involved in reminding patients about medication, diet, physical activity, or healthcare visits?

b. What forms of family support are most helpful for patients?

c. What family roles are most important in supporting daily hypertension self-care?

*Core Question:*

B2. What are the main challenges families face in supporting patient behavior change?

*Optional probes:*

a. What makes it difficult for families to provide consistent support?

b. How do families manage stress or competing responsibilities while supporting the patient?

**Socio-Cultural Factors**

*Core Question:*

C1. How do social or cultural norms influence hypertension self-management?

*Optional probes:*

a. Are there local cultural practices (e.g., Balinese ceremonies, dietary habits, or traditional medicine) that affect self-care?

b. Do patients use traditional treatments or other non-medical practices? If yes, why?

c. How do family beliefs about medical care and traditional care influence patient decisions?

*Core Question:*

C2. How do patients and families perceive the need for changes in diet or lifestyle to support hypertension self-care?

*Optional probes:*

a. What lifestyle changes are considered easy or difficult to implement?

b. What local habits or customs may support or hinder these changes?

*Core Question:*

C3. What cultural beliefs or local customs should be considered in developing the self-care bundle?

*Optional probes:*

a. What cultural values should be respected in the module?

b. What messages would make the module more acceptable for patients and families?

*Core Question:*

C4. What barriers or difficulties do patients and families experience in implementing the self-care bundle at home?

**Healthcare Service Factors**

*Core Question:*

D1. How do patients evaluate the role of nurses and healthcare staff in supporting self-care?

*Optional probes:*

a. Do patients feel that they receive adequate education, counseling, or follow-up?

b. What type of support from nurses or healthcare staff is most helpful?

*Core Question:*

D2. How do existing healthcare services or policies support patients in performing self-care?

*Optional probes:*

a. How do patients perceive current services in enabling family involvement?

b. What are patients’ expectations regarding healthcare support for hypertension self-care?

**Integrated Self-Care Bundle (PCN-Based)**

*Core Question:*

E1. What are patients’ and families’ views on the proposed integrated self-care bundle, including blood pressure monitoring, lifestyle education, medication adherence, and motivation for routine healthcare visits?

*Optional probes:*

a. Which components are most useful for daily self-care?

b. Which components may be difficult to implement?

c. How should the module be designed so that it is easy to understand and use?

*Core Question:*

E2. What additional components should be included in the module?

*Optional probes:*

a. Are there additional topics related to local habits, culture, or family roles that should be included?

b. What information do patients and families still need to support hypertension self-care?

*Core Question:*

E3. How can the integrated approach make hypertension self-care easier for patients and families?

*Optional probes:*

a. How can the module help patients communicate with nurses during routine visits?

b. How can family members use the module to support patients at home?

**Self-Management Abilities**

*Core Question:*

F1. How do patients monitor their blood pressure at home or at healthcare facilities?

*Optional probes:*

a. What difficulties do patients experience in monitoring blood pressure?

b. What support is needed for patients who do not have a blood pressure device at home?

*Core Question:*

F2. How do patients manage diet, medication, physical activity, sleep, and stress?

*Optional probes:*

a. What foods do patients avoid or consume more to maintain blood pressure?

b. Do patients find it difficult to follow medication schedules? How does the family help?

c. Do patients exercise regularly? What challenges do they experience?

d. Do patients feel they get enough sleep?

e. How do patients manage stress, and does stress affect their blood pressure?

*Core Question:*

F3. What are the biggest challenges in maintaining adherence to hypertension self-care?

*Optional probes:*

a. What challenges are related to diet, medication, blood pressure monitoring, physical activity, or healthcare visits?

b. What family support is needed to overcome these challenges?

**Suggestions for Improving Hypertension Self-Care Support**

G1. What recommendations do patients and families have to improve the hypertension self-care program?

**FGD 2: Nurses and Healthcare Providers**

Participant Information:

Name: ___________________________

Role: Nurse / Primary Healthcare Staff

Institution / Organization: ___________________________

Instructions:

This guide was used flexibly during the FGD. The main questions were used to guide discussion, while the probes were optional and used only when further clarification or elaboration was needed. The order of questions could be adjusted according to the flow of discussion. Before questions concerning the proposed bundle, the moderator provided a brief neutral overview of the four candidate domains without presenting preferred responses.

**Patient Factors: Knowledge and Self-Efficacy**

*Core Question:*

A1. How do nurses or healthcare providers assess patients’ knowledge about hypertension self-care?

*Optional probes:*

a. How have existing programs supported patient self-care?

b. What barriers do patients face in understanding hypertension self-care?

c. What strategies do nurses use to overcome these barriers?

*Core Question:*

A2. How do nurses or healthcare providers support patients’ self-efficacy in performing hypertension self-care?

*Optional probes:*

a. To what extent are patients able to perform self-care independently?

b. What strategies help patients become more confident in managing hypertension?

c. What factors reduce patients’ confidence or motivation?

**Family Factors: Coping, Support, Role**

*Core Question:*

B1. How do nurses perceive the role of family in supporting patient self-care?

*Optional probes:*

a. How are families involved in medication reminders, diet, physical activity, monitoring, or healthcare visits?

b. What family roles are most helpful in supporting hypertension self-care?

*Core Question:*

B2. To what extent are families involved in existing hypertension care programs?

*Optional probes:*

a. What facilitates family involvement?

b. What limits family involvement in routine care?

*Core Question:*

B3. How do families cope with the demands of supporting patients with hypertension?

*Optional probes:*

a. What coping strategies are helpful?

b. What coping patterns may obstruct patient self-care?

**Socio-Cultural Factors**

*Core Question:*

C1. How do social or cultural practices influence hypertension self-care?

*Optional probes:*

a. How do cultural habits, such as diet, ceremonies, or traditional medicine, affect self-care?

b. What steps do healthcare centers take to address cultural barriers?

c. How does Balinese culture influence patient perception and adherence to self-care?

*Core Question:*

C2. What cultural or socio-economic barriers may hinder adoption of the self-care bundle?

*Optional probes:*

a. What barriers are commonly encountered in primary care?

b. What local values or practices should be considered when implementing the bundle?

**Healthcare Service Factors**

*Core Question:*

D1. How do nurses evaluate their own competencies in supporting hypertension self-care?

*Optional probes:*

a. Do nurses have sufficient training to educate and monitor patients with hypertension?

b. What strategies are used to improve nurse competence?

c. What challenges exist due to limited resources, workload, or time?

*Core Question:*

D2. How do existing healthcare policies or service systems support patient self-care?

*Optional probes:*

a. What policies or service routines currently support hypertension self-care?

b. What changes are needed to improve nurse effectiveness in supporting patients and families?

**Integrated Self-Care Bundle (PCN-Based)**

*Core Question:*

E1. How acceptable, feasible, and relevant is the proposed integrated self-care bundle for supporting hypertension self-care in primary care?

*Optional probes:*

a. Which components are most relevant for routine primary care?

b. Which components may be difficult to implement?

c. How can the bundle be adapted to fit primary care workflow?

*Core Question:*

E2. What additions or changes are needed to improve the usability of the bundle?

*Optional probes:*

a. What should be improved in the patient module?

b. What should be improved in the nurse module?

c. What information, checklist, or documentation format would be most useful?

*Core Question:*

E3. Which cultural or local habits should be considered to improve acceptance and use of the bundle?

*Optional probes:*

a. What culturally sensitive messages should be included?

b. How can nurses discuss lifestyle modification without dismissing patients’ values or daily realities?

**Self-Management Abilities**

*Core Question:*

F1. How do nurses assist patients in monitoring blood pressure?

*Optional probes:*

a. What challenges do patients experience in blood pressure monitoring?

b. How do nurses respond when patients do not have a home blood pressure device?

*Core Question:*

F2. What challenges do nurses face in supporting diet, medication adherence, physical activity, sleep, and stress management?

*Optional probes:*

a. What difficulties arise in helping patients follow dietary recommendations?

b. What challenges occur in helping patients follow medication schedules?

c. How do nurses support patients in maintaining physical activity?

d. How do nurses address sleep problems and stress that may affect self-care?

*Core Question:*

F3. What are the biggest challenges for nurses and families in supporting patient adherence and monitoring?

*Optional probes:*

a. What barriers are related to patient behavior?

b. What barriers are related to family support?

c. What barriers are related to healthcare service limitations?

**Suggestions for Improving Hypertension Self-Care Support**

G1. What recommendations should be implemented to enhance the self-care program in terms of policy, nurse role, or family support?
