# Supplementary material for: A dual-module person-centered nursing self-care bundle for hypertension management: development and expert validation
Source: BMC Nurs. 2026 Sep 26;25:987. doi: 10.1186/s12912-026-05377-2 (PMC13673632; doi:10.1186/s12912-026-05377-2)
Supplement: Supplementary file 2 [file 12912_2026_5377_MOESM2_ESM.docx]

**Supplementary File 2.** Expert Validation Questionnaire – English Version

**Expert Validation Questionnaire for the Dual-Module PCN-Based Self-Care Bundle**

Expert Information:

Name: ___________________________

Position /Title: ___________________________

Institution: ___________________________

Instructions:

Please evaluate each item using the following four-point suitability scale:

4 = Appropriate without revision

3 = Appropriate with minor revision

2 = Requires major revision

1 = Not appropriate

Please place a check mark (√) in the column that best represents your evaluation of each item. Comments or suggestions may be provided to explain the rating and to guide module refinement.

| **Assessment Component** | **Indicator** | **Appropriate without revision** | **Appropriate with minor revision** | **Requires major revision** | **Not appropriate** | **Comments / Suggestions** |
| --- | --- | --- | --- | --- | --- | --- |
| Content | Alignment of material with standards |  |  |  |  |  |
|  | Basic competency |  |  |  |  |  |
|  | Accuracy of material |  |  |  |  |  |
|  | Supporting learning materials |  |  |  |  |  |
|  | Currency of material |  |  |  |  |  |
| Presentation | Presentation technique |  |  |  |  |  |
|  | Presentation support |  |  |  |  |  |
|  | Learning presentation |  |  |  |  |  |
|  | Completeness of presentation |  |  |  |  |  |
| Language | Conciseness |  |  |  |  |  |
|  | Communicativeness |  |  |  |  |  |
|  | Dialogic and interactive language |  |  |  |  |  |
|  | Suitability for respondents’ developmental level |  |  |  |  |  |
|  | Coherence and logical flow |  |  |  |  |  |
|  | Use of terms, symbols, or icons |  |  |  |  |  |
| Graphic design | Physical size of the module |  |  |  |  |  |
|  | Cover design |  |  |  |  |  |
|  | Internal layout design |  |  |  |  |  |
| Health promotion media | Criteria as health promotion media |  |  |  |  |  |
|  | Effectiveness and creativity as health promotion media |  |  |  |  |  |
