# Supplementary material for: A dual-module person-centered nursing self-care bundle for hypertension management: development and expert validation
Source: BMC Nurs. 2026 Sep 26;25:987. doi: 10.1186/s12912-026-05377-2 (PMC13673632; doi:10.1186/s12912-026-05377-2)
Supplement: Supplementary file 3 [file 12912_2026_5377_MOESM3_ESM.docx]

**Supplementary Table 1.** Strategic Issues Informing Module Development

| **Strategic area** | **Key empirical findings** | **Strategic issue identified** | **Implication for dual-module development** |
| --- | --- | --- | --- |
| Patient factors | Low knowledge and low self-efficacy were identified among patients with hypertension. | Patients had limited understanding and confidence in performing comprehensive hypertension self-care. | The patient module needed simple explanations, practical self-care guidance, confidence-building activities, and structured self-monitoring tools. |
| Family factors | Family coping, support, and caregiving roles were not optimal. | Family involvement was present but insufficient to support sustained self-care behaviors. | The patient module needed family support guidance, while the nurse module needed prompts to assess and strengthen family involvement during follow-up. |
| Socio-cultural factors | Health-related habits, treatment beliefs, and illness perceptions were not fully supportive of self-care. | Cultural routines, food practices, medication beliefs, and illness perceptions affected self-care consistency. | Both modules needed culturally sensitive messages, particularly regarding diet, traditional practices, medication beliefs, and daily routines. |
| Healthcare service factors | Professional support and service policy support were perceived as limited. | Continuous self-care support was constrained by service workload, resources, and limited structured follow-up mechanisms. | The nurse module needed structured guidance for education, monitoring, documentation, feedback, and follow-up in routine primary care. |
| Candidate bundle components | The combined candidate-domain construct indicated a need for more integrated and sustained self-care support across the four predefined domains. | Core self-care support components were not yet implemented in an integrated and sustained manner. | The quantitative phase supported retaining the four predefined domains for contextual refinement during module development. |
| Self-management ability | Blood pressure monitoring, diet adherence, rest and sleep, physical activity, medication adherence, and stress management were suboptimal. | Patients had difficulty implementing comprehensive and consistent hypertension self-management. | The patient module needed self-care checklists and daily records, while the nurse module needed evaluation indicators for monthly review. |
| PCN integration | Self-care support needed to reflect patients’ values, preferences, family context, and communication needs. | Conventional education alone was insufficient to support individualized and sustained self-care. | PCN principles were integrated into both modules through shared decision-making, holistic assessment, supportive communication, and individualized follow-up. |

**Supplementary Table 2**. FGD Findings and Translation Into Module Components

| **FGD-derived issue** | **Key stakeholder findings** | **Translation into the patient module** | **Translation into the nurse module** |
| --- | --- | --- | --- |
| Fragmented self-care practices | Patients tended to focus mainly on medication use and did not consistently perform blood pressure monitoring, diet control, physical activity, stress management, and routine follow-up. | Integrated self-care guidance covering blood pressure monitoring, lifestyle modification, medication adherence, stress management, and healthcare visits. | Monthly review guide to assess all bundle components, not only medication use. |
| Low confidence in self-monitoring | Some patients were not confident in measuring blood pressure independently and preferred to check it at healthcare facilities or with other people. | Blood pressure monitoring guidance, recording sheets, and alternatives for patients without home blood pressure devices. | Counseling prompts to identify barriers to self-monitoring and provide feasible alternatives. |
| Medication concerns and treatment fatigue | Patients expressed concerns about long-term antihypertensive medication and sometimes stopped medication when symptoms improved. | Plain-language information on medication adherence, common concerns, and questions to discuss with healthcare providers. | Guidance for addressing medication beliefs, correcting misconceptions, reinforcing adherence, and documenting medication-related concerns. |
| Limited family involvement | Families commonly helped with healthcare visits and medication reminders, but were less involved in diet, physical activity, emotional support, and long-term monitoring. | Family support section with concrete roles in daily care, including diet, activity, emotional support, and monitoring. | Family involvement assessment and family-centered counseling prompts during follow-up. |
| Time constraints among family members | Family members reported difficulty being consistently involved due to work and daily responsibilities. | Practical family support strategies that can be adapted to time availability and shared among family members. | Guidance for helping patients and families agree on realistic support roles. |
| Socio-cultural dietary challenges | Traditional ceremonies, religious holidays, and local food practices influenced salt, fat, and food intake patterns. | Culturally sensitive dietary strategies, including portion control and healthier choices during social or cultural events. | Counseling guide for negotiating lifestyle changes while respecting patient values and cultural context. |
| Use of herbal or traditional remedies | Some patients used herbal remedies as complementary practices while continuing medical treatment. | Guidance encouraging patients to discuss herbal or traditional practices with healthcare providers. | Prompts for nurses to explore treatment beliefs without judgment and provide safe, evidence-informed advice. |
| Limited continuity of care | Healthcare services remained facility-centered, and continuous monitoring outside visits was difficult due to limited resources. | Patient-held self-care records and checklists to support communication during monthly visits. | Structured follow-up documentation, evaluation indicators, and feedback guidance for routine primary care visits. |
| Limited hypertension-specific nursing tools | Nurses reported that hypertension education was often delivered as part of general non-communicable disease services. | Clear patient education materials that can be reviewed repeatedly at home. | Operational guide with session objectives, key education points, monitoring flow, and documentation tools. |
| Need for simple and attractive materials | Patients, families, and providers supported a clear, structured, easy-to-use self-care guide. | Patient-friendly language, checklist format, self-care records, and spaces for barriers and questions. | Practical implementation guide with concise instructions, evaluation forms, and follow-up planning tools. |

**Supplementary Table 3.** Evidence-to-module mapping

| **Source of evidence** | **Key input identified** | **Translation into the patient module** | **Translation into the nurse module** |
| --- | --- | --- | --- |
| Prior empirical findings | Four theory-informed candidate domains were retained and supported by the preceding quantitative needs-assessment: self-monitoring assistance, lifestyle-modification education, medication-regimen monitoring, and motivation for routine healthcare visits. | Self-care education, blood pressure monitoring sheets, medication reminders, lifestyle guidance, and visit preparation checklist. | Nurse guidance for education delivery, monitoring indicators, evaluation checklist, and follow-up documentation. |
| Patient and family FGD | Patients and families needed practical, simple, and culturally appropriate guidance for daily hypertension care. | Simplified language, practical examples, family support tips, warning signs, and daily self-care checklist. | Counseling prompts to explore patient preferences, barriers, family support, and home-based self-care practices. |
| Healthcare provider FGD | Nurses needed structured tools to educate, monitor, and evaluate patient self-care during primary care visits. | Patient-held record to facilitate communication with nurses during visits. | Session objectives, education scripts, monitoring forms, evaluation points, and documentation tools. |
| Expert consultation | The module needed stronger alignment with PCN principles and clearer behavioral strategies. | Goal-setting forms, self-monitoring activities, and patient reflection sections. | Shared decision-making prompts, feedback mechanisms, and person-centered follow-up guidance. |
| Expert validation | Most items met the prespecified agreement threshold; selected content and presentation items required revision, while qualitative expert comments informed refinements across the five assessment domains. | Readability, layout, visual clarity, and user-friendly instructions were refined. | Clearer nurse instructions, structured monitoring flow, and improved documentation format. |

**Supplementary Table 4.** Item-level expert assessment results of the patient module (n = 7)

| **Validation aspect** | **Item** | **Rating 3 (n)** | **Rating 4 (n)** | **Ratings 3 or 4 (n)** | **Item-level agreement** | **Interpretation** |
| --- | --- | --- | --- | --- | --- | --- |
| Content | Alignment of material with standards | 0 | 5 | 5 | 0.71 | Revised |
|  | Basic competency | 0 | 6 | 6 | 0.86 | Valid |
|  | Accuracy of material | 1 | 6 | 7 | 1.00 | Valid |
|  | Supporting learning materials | 1 | 5 | 6 | 0.86 | Valid |
|  | Currency of material | 0 | 7 | 7 | 1.00 | Valid |
| Presentation | Presentation technique | 1 | 6 | 7 | 1.00 | Suitable |
|  | Presentation support | 0 | 7 | 7 | 1.00 | Suitable |
|  | Learning presentation | 0 | 7 | 7 | 1.00 | Suitable |
|  | Completeness of presentation | 0 | 5 | 5 | 0.71 | Revised |
| Language | Conciseness | 1 | 6 | 7 | 1.00 | Suitable |
|  | Communicativeness | 0 | 6 | 6 | 0.86 | Suitable |
|  | Dialogic and interactive language | 0 | 6 | 6 | 0.86 | Suitable |
| Language | Suitability for respondents’ developmental level | 0 | 7 | 7 | 1.00 | Suitable |
|  | Coherence and logical flow | 0 | 7 | 7 | 1.00 | Suitable |
|  | Use of terms, symbols, or icons | 1 | 6 | 7 | 1.00 | Suitable |
| Graphic design | Physical size of the module | 0 | 7 | 7 | 1.00 | Suitable |
|  | Cover design | 2 | 4 | 6 | 0.86 | Suitable |
|  | Internal layout design | 0 | 7 | 7 | 1.00 | Suitable |
| Health promotion media | Criteria as health promotion media | 1 | 6 | 7 | 1.00 | Suitable |
|  | Effectiveness and creativity as health promotion media | 0 | 7 | 7 | 1.00 | Suitable |

**Note:** For the content domain, ratings were interpreted as item-level content validity index (I-CVI). For presentation, language, graphic design, and health-promotion media suitability domains, the same agreement calculation was used to describe expert agreement regarding domain-specific suitability rather than content validity in the strict psychometric sense. “Valid” indicates that the item met the prespecified agreement threshold (I-CVI ≥0.86).

**Supplementary Table 5.** Item-level expert assessment results of the nurse module (n = 7)

| **Validation aspect** | **Item** | **Rating 3 (n)** | **Rating 4 (n)** | **Ratings 3 or 4 (n)** | **Item-level agreement** | **Interpretation** |
| --- | --- | --- | --- | --- | --- | --- |
| Content | Alignment of material with standards | 2 | 5 | 7 | 1.00 | Valid |
|  | Basic competency | 0 | 6 | 6 | 0.86 | Valid |
|  | Accuracy of material | 1 | 6 | 7 | 1.00 | Valid |
|  | Supporting learning materials | 0 | 5 | 5 | 0.71 | Revised |
|  | Currency of material | 0 | 7 | 7 | 1.00 | Valid |
| Presentation | Presentation technique | 0 | 7 | 7 | 1.00 | Suitable |
|  | Presentation support | 0 | 6 | 6 | 0.86 | Suitable |
|  | Learning presentation | 0 | 7 | 7 | 1.00 | Suitable |
|  | Completeness of presentation | 1 | 5 | 6 | 0.86 | Suitable |
| Language | Conciseness | 0 | 7 | 7 | 1.00 | Suitable |
|  | Communicativeness | 1 | 6 | 7 | 1.00 | Suitable |
|  | Dialogic and interactive language | 0 | 6 | 6 | 0.86 | Suitable |
|  | Suitability for respondents’ developmental level | 0 | 7 | 7 | 1.00 | Suitable |
|  | Coherence and logical flow | 0 | 7 | 7 | 1.00 | Suitable |
|  | Use of terms, symbols, or icons | 0 | 7 | 7 | 1.00 | Suitable |
| Graphic design | Physical size of the module | 0 | 7 | 7 | 1.00 | Suitable |
|  | Cover design | 0 | 7 | 7 | 1.00 | Suitable |
|  | Internal layout design | 0 | 7 | 7 | 1.00 | Suitable |
| Health promotion media | Criteria as health promotion media | 0 | 6 | 6 | 0.86 | Suitable |
|  | Effectiveness and creativity as health promotion media | 1 | 6 | 7 | 1.00 | Suitable |

**Note:** For the content domain, ratings were interpreted as item-level content validity index (I-CVI). For presentation, language, graphic design, and health-promotion media suitability domains, the same agreement calculation was used to describe expert agreement regarding domain-specific suitability rather than content validity in the strict psychometric sense. “Valid” indicates that the item met the prespecified agreement threshold (I-CVI ≥0.86).

**Supplementary Table 6.** Expert Consultation Feedback and Module Refinement

| **Area reviewed** | **Expert feedback** | **Refinement in the patient module** | **Refinement in the nurse module** |
| --- | --- | --- | --- |
| Content relevance | The modules needed to clearly reflect the four core bundle components and remain focused on hypertension self-care. | Content was organized around self-monitoring, lifestyle modification, medication adherence, and routine healthcare visits. | Nurse guidance was aligned with the same four bundle components for education, monitoring, and evaluation. |
| PCN alignment | The modules needed stronger integration of patient preferences, values, barriers, and shared decision-making. | Reflection spaces were added for patients to record barriers, questions, preferences, and goals for discussion during visits. | Counseling prompts were added to guide nurses in exploring patient values, barriers, preferences, and readiness for change. |
| Readability | Technical terms needed to be simplified for patient use. | Language was revised into simpler, patient-friendly wording with practical examples. | Professional terms were retained where necessary but supported with operational instructions for implementation. |
| Family involvement | Family support needed to be made more concrete and practical. | A family support section was strengthened with specific roles in diet, medication reminders, activity, emotional support, and visit preparation. | Nurses were guided to assess family involvement and provide family-centered education during follow-up. |
| Cultural appropriateness | Lifestyle guidance needed to be realistic and sensitive to local cultural practices. | Cultural considerations were added, including practical strategies for maintaining self-care during ceremonies, holidays, and social events. | Nurses were guided to discuss lifestyle-modification without dismissing patients’ cultural values or daily realities. |
| Self-monitoring tools | Patients needed clearer guidance for recording blood pressure and self-care practices. | Daily self-care checklists, blood pressure records, and spaces for barriers or symptoms were strengthened. | Nurses were guided to review patient records, identify patterns, and provide individualized feedback. |
| Medication adherence | Medication-related concerns and misconceptions needed to be addressed directly. | Patient-friendly explanations and questions about medication use were added. | Nurses were guided to explore medication beliefs, clarify misconceptions, and document adherence barriers. |
| Follow-up process | The link between home-based self-care and monthly healthcare visits needed to be clearer. | Patients were instructed to bring completed records and write topics they wished to discuss during routine visits. | Nurses were guided to review completed checklists, discuss patient-selected topics, provide feedback, and formulate follow-up plans. |
| Documentation | The modules needed to support monitoring and continuity of care. | Patient records were structured to document self-care activities, barriers, questions, and progress. | Documentation forms were strengthened to record evaluation results, feedback, and agreed follow-up plans. |
| Feasibility | The modules needed to be usable in routine primary care without adding excessive workload. | Patient activities were simplified and structured into practical daily and monthly tasks. | Nurse activities were organized into concise review, feedback, and documentation steps for routine visits. |

**Supplementary Table 7.** Expert feedback and refinement of the dual modules

| **Area refined** | **Expert feedback** | **Refinement in the patient module** | **Refinement in the nurse module** |
| --- | --- | --- | --- |
| Competency orientation | The modules should not only evaluate knowledge but also support attitude and self-care actions. | Self-care tasks, daily checklists, and patient reflection sections were strengthened. | Nurse evaluation indicators were clarified to assess self-care implementation, not only patient knowledge. |
| Home-based practice | The module needed more practical tasks that patients could perform at home. | Practical home-based activities were added for blood pressure monitoring, diet, activity, medication adherence, and stress management. | Nurses were guided to review completed tasks and provide feedback during routine visits. |
| Medication information | Medication concerns, long-term use, and complementary or traditional remedies needed clearer explanation. | Medication adherence guidance and spaces for medication-related questions were revised and expanded. | Counseling prompts were added to help nurses explore medication beliefs and clarify misconceptions. |
| Language | Some terms needed to be simplified for lay users. | Technical terms were simplified and supported with clearer explanations. | Operational instructions were made more concise and easier to apply in practice. |
| Interactivity | The patient module needed to be more interactive rather than only informational. | Checklists, self-monitoring sheets, goal-setting spaces, and questions for monthly visits were strengthened. | Nurses were guided to use patient records as the basis for discussion, feedback, and follow-up planning. |
| Follow-up mechanism | The modules needed clearer guidance for patients who miss routine visits or experience difficulties. | Follow-up planning and sections for barriers or problems were clarified. | Nurse follow-up actions and documentation procedures were strengthened. |
| Visual and media quality | The modules were considered suitable as health-promotion media but needed consistent layout and readability. | Layout, readability, and user-friendly instructions were refined. | Monitoring flow, documentation format, and checklist review sections were refined. |

**Supplementary Table 8.** Item-level audit trail for low-agreement items and post-revision review

| **Module** | **Validation domain and item** | **Pre-revision agreement** | **Item-specific expert concern** | **Revision made** | **Location in revised module** | **Post-revision outcome** |
| --- | --- | --- | --- | --- | --- | --- |
| Patient module | Content – Alignment of material with standards | 0.71 | Experts requested clearer alignment between the module content and the four predefined self-care domains. | The relationship between the module content and the four predefined self-care domains was made more explicit, and the integrated self-care activities were aligned with the PCN-based bundle components. | Chapter 7: Practice of the Integrated PCN-Based Self-Care Bundle | The experts confirmed that the requested revision had been addressed during the qualitative post-revision check; no numerical re-rating was performed. |
| Patient module | Presentation – Completeness of presentation | 0.71 | Experts requested a more complete presentation of practical, recording, feedback, and follow-up components. | Self-care recording components, monthly review, feedback, and follow-up planning were strengthened and presented more explicitly. | Chapters 7 and 8, including Feedback, Monthly Monitoring, and Follow-Up Planning | The experts confirmed that the requested revision had been addressed during the qualitative post-revision check; no numerical re-rating was performed. |
| Nurse module | Content – Supporting learning materials | 0.71 | Experts requested more operational supporting materials to guide nurse-led implementation, monitoring, feedback, documentation, and follow-up. | Implementation guidance, monitoring and review procedures, feedback and follow-up guidance, and supporting forms/tools were expanded and operationalized. | Chapter 7: Implementation Guide for the Integrated PCN-Based Self-Care Bundle; Chapter 8: Feedback and Follow-Up Planning; and Appendices | The experts confirmed that the requested revision had been addressed during the qualitative post-revision check; no numerical re-rating was performed. |

**Supplementary Table 9.** Theoretical, empirical, and contextual rationale for the four predefined self-care support domains

| **Candidate domain** | **Theoretical and evidence-based rationale** | **Contextual need addressed** | **Translation into the modules** |
| --- | --- | --- | --- |
| Self-monitoring assistance | Hypertension self-management requires regular blood-pressure monitoring, recognition of warning signs, and interpretation of monitoring results with professional support. (1–3) | Inconsistent blood-pressure monitoring and limited recording of home-based self-care activities. | Blood-pressure records, symptom monitoring, self-care checklists, identification of monitoring barriers, and nurse review. |
| Lifestyle-modification education | Long-term hypertension care requires sustained dietary modification, physical activity, adequate rest, stress management, and adaptation to patients’ sociocultural context. (4,5) | Difficulty maintaining lifestyle changes, particularly within family routines and cultural or social events. | Practical lifestyle guidance, culturally appropriate examples, family-support activities, daily checklists, and negotiated lifestyle goals. |
| Medication-regimen monitoring | Effective hypertension control requires adherence to prescribed medication, understanding of the regimen, identification of barriers, and continued review. (3,5,6) | Missed medication, uncertainty regarding medication routines, and family involvement limited mainly to reminders. | Medication schedules, adherence records, documentation of medication-related barriers, family reminders, and nurse-led review. |
| Motivation for routine healthcare visits | Continuity of hypertension management requires scheduled follow-up, professional feedback, monitoring of progress, and timely response to emerging problems. (6,7) | Irregular follow-up and limited continuity between patients’ home-based self-care and routine clinical review. | Visit reminders, visit-preparation notes, recording of questions and barriers, nurse feedback, and documented follow-up planning. |

**Note:** In the preceding quantitative needs assessment, the four predefined self-care support domains were examined collectively as indicators of a candidate-domain construct. The findings supported the contextual relevance and retention of these domains and informed their subsequent refinement through stakeholder consultation and module development. Detailed methods, statistical analyses, effect estimates, and extended interpretation of the needs assessment are not reproduced here and are reserved for a separate manuscript currently under peer review.

**Supplementary Table 10.** Characteristics of focus group discussion participants

| **Characteristic** | **Patients**  **(n = 4)** | **Family caregivers (n = 4)** | **Frontline healthcare providers (n = 5)** | **Primary healthcare leaders (n = 3)** |
| --- | --- | --- | --- | --- |
| Age, median (range), years | 51.0 (46–58) | 28.5 (25–35) | 38.0 (26–46) | 54.0 (51–57) |
| Duration of hypertension, median (range), years | 6.0 (2–7) | Not applicable | Not applicable | Not applicable |
| Duration of caregiving, median (range), years | Not applicable | 5.0 (5–7) | Not applicable | Not applicable |
| Relevant professional experience, median (range), years | Not applicable | Not applicable | 7.0 (1–17) | 25.0 (4–28) |
| Professional composition | Not applicable | Not applicable | 5 nurses | 3 physicians |
| Service composition | Not applicable | Not applicable | Hypertension, NCD, or older-person program providers | 2 center heads; 1 representative of a center head |
| Main stakeholder role | Person living with hypertension | Family caregiver | Primary care nursing provider | Primary healthcare service leader |

Note: NCD, non-communicable disease

**Supplementary Table 11.** Characteristics and roles of the preliminary consultants involved in initial module refinement (n = 3)

| **Consultant code** | **Professional role and qualification** | **Primary area of expertise** | **Relevant experience, years** | **Selection criteria** | **Contribution to preliminary module refinement** | **Relationship to the authors** |
| --- | --- | --- | --- | --- | --- | --- |
| C1 | Clinical cardiovascular nurse and educator with a doctoral degree in Nursing and a specialist qualification in Medical-Surgical Nursing | Cardiovascular nursing, hypertension care, and clinical self-care management | 20 | Relevant postgraduate specialist qualification; at least five years of academic or clinical experience; demonstrated understanding of self-care and hypertension management | Advised on development of the self-care bundle according to patient needs, reviewed the completeness and clinical relevance of the bundle components, and provided recommendations regarding implementation in clinical practice | Affiliated with an institution different from those of the authors and the other preliminary consultants; no supervisory, line-management, authorship, decision-making, or other study-related role. |
| C2 | Nursing academic with a doctoral qualification in Nursing | Cardiovascular nursing, evidence-based hypertension care, and Person-Centered Nursing | 10 | Relevant master’s specialist or doctoral qualification; at least five years of academic or clinical experience; demonstrated understanding of self-care, PCN, and hypertension management | Advised on the structure of the self-care bundle, integration of evidence-based practice in hypertension care, and incorporation of PCN principles into the modules | Affiliated with an institution different from those of the authors and the other preliminary consultants; no supervisory, line-management, authorship, decision-making, or other study-related role. |
| C3 | Mental health nursing academic; Master of Nursing with a specialist qualification in Mental Health Nursing | Psychosocial care, behavioral support, patient motivation, and self-care management | 8 | Relevant master’s specialist or doctoral qualification; at least five years of academic or clinical experience; demonstrated understanding of self-care, PCN, and hypertension management | Advised on integration of psychosocial components, identification of psychological barriers to self-care, and strategies to support patient motivation during implementation | Affiliated with an institution different from those of the authors and the other preliminary consultants; no supervisory, line-management, authorship, decision-making, or other study-related role. |

**Note:** They did not assign numerical ratings and were not included in the separate seven-member formal expert-validation panel.

**Supplementary Table 12.** Characteristics of the formal expert-validation panel (n = 7)

| **Expert code** | **Professional background and qualification** | **Primary area of expertise** | **Relevant experience, years** | **Current role and setting** | **Relationship to the research team and independence** |
| --- | --- | --- | --- | --- | --- |
| E1 | Doctoral degree in Nursing | Chronic disease nursing and self-care management | 10 | Nursing academic and researcher at a higher education institution | Independent of the research team; no authorship, supervisory, line-management, or involvement in study design, data collection, or analysis |
| E2 | Doctoral degree in Nursing | Community nursing and primary healthcare | 8 | Community nursing academic and researcher at a higher education institution | Independent of the research team; no authorship, supervisory, line-management, or involvement in study design, data collection, or analysis |
| E3 | Master of Nursing with specialist qualification in Medical-Surgical Nursing | Cardiovascular nursing and hypertension care | 5 | Medical-surgical nursing academic and clinical educator at a higher education institution | Independent of the research team; no authorship, supervisory, line-management, or involvement in study design, data collection, or analysis |
| E4 | Master of Nursing with specialist qualification in Community Nursing | Community nursing, health promotion, and patient education | 5 | Community nursing academic and clinical educator at a higher education institution | Independent of the research team; no authorship, supervisory, line-management, or involvement in study design, data collection, or analysis |
| E5 | Registered nurse | Non-communicable disease management and hypertension services | 7 | Primary care nurse responsible for the non-communicable disease program at a public health centre | Independent of the research team; involved only as a formal expert validator and had no role in study design, data collection, or analysis |
| E6 | Specialist physician in Cardiology and Vascular Medicine | Cardiovascular medicine, hypertension diagnosis, and clinical management | 15 | Cardiologist providing cardiovascular services in a hospital setting | Independent of the research team; involved only as a formal expert validator and had no role in study design, data collection, or analysis |
| E7 | General practitioner | Primary care medicine and hypertension management | 8 | General practitioner providing hypertension and chronic disease services at a public health centre | Independent of the research team; involved only as a formal expert validator and had no role in study design, data collection, or analysis |

**Supplementary References**

Supplementary references supporting the theoretical, empirical, and contextual rationale presented in Supplementary Table 9 are provided below.

1.	Aungsuroch Y, Polsook R, Anuruang S, Sitha, Phongphibool, Gunawan J, et al. How a Self ‑ Management Program Affects Blood Pressure Among Indonesians with Hypertension : A Quasi ‑ Experimental Study. Iran J Nurs Midwifery Res. 2022;27(3):229–35.

2.	Sanchez M, Hara-Hubbard KK, Baquero B. Evaluating the Effectiveness of a Patient-Centered, Nonphysician Led Self-Monitoring Blood Pressure Program in a Rural Federally Qualified Health Center. J Public Heal Manag Pract. 2024;30(5):S167–74.

3.	Kurt D, Gurdogan EP. The effect of self-management support on knowledge level, treatment compliance and self-care management in patients with hypertension. Aust J Adv Nurs. 2022;39(3):14–23.

4.	Putri SE, Rekawati E, Wati DNK. Effectiveness of self-management on adherence to self-care and on health status among elderly people with hypertension. J Public health Res. 2021;10:75–81.

5.	Setiadi AP, Febriandini A, Trinanda E, Aryaguna W, Mutho’atin Chusna I, Nurlaili Y, et al. Knowing the gap: medication use, adherence and blood pressure control among patients with hypertension in Indonesian primary care settings. PeerJ. 2022;10:1–18.

6.	Roberti J, Vita T, Piastrella J, Porley C, Pereyra L, Diez M, et al. Care bundle to reduce readmission in patients with heart failure: A modified Delphi consensus panel in Argentina. BMJ Open. 2020;10(12):1–7.

7.	Mills KT, Peacock E, Chen J, Zimmerman A, Brooks K, He H, et al. Implementation of Multifaceted Patient-Centered Treatment Strategies for Intensive Blood Pressure Control (IMPACTS): Rationale and design of a cluster-randomized trial. Am Heart J. 2020;230(January):13–24.
