# Supplementary material for: A dual-module person-centered nursing self-care bundle for hypertension management: development and expert validation
Source: BMC Nurs. 2026 Sep 26;25:987. doi: 10.1186/s12912-026-05377-2 (PMC13673632; doi:10.1186/s12912-026-05377-2)
Supplement: Supplementary file 4 [file 12912_2026_5377_MOESM4_ESM.docx]

**Manuscript:** A Dual-Module Person-Centered Nursing Self-Care Bundle for Hypertension Management: Development and Expert Validation

**Consolidated criteria for reporting qualitative studies (COREQ): 32-item checklist**

Developed from:

Tong A, Sainsbury P, Craig J. Consolidated criteria for reporting qualitative research (COREQ): a 32-item checklist for interviews and focus groups. *International Journal for Quality in Health Care*. 2007. Volume 19, Number 6: pp. 349 – 357

| **No. Item** | **Guide questions/description** | **Reported on Page #** | |
| --- | --- | --- | --- |
| **Domain 1: Research team and reﬂexivity** |  |  | |
| *Personal Characteristics* |  |  | |
| 1. Inter viewer/facilitator | Which author/s conducted the interview or focus group? | Page 9, NPARD, the principal researcher, moderated both FGDs. | |
| 2. Credentials | What were the researcher’s credentials? E.g. PhD, MD | Page 9 | |
| 3. Occupation | What was their occupation at the time of the study? | Page 9, NPARD was a doctoral nursing student and the principal researcher | |
| 4. Gender | Was the researcher male or female? | Page 9, Female | |
| 5. Experience and training | What experience or training did the researcher have? | Page 9, The moderator had completed formal training in qualitative research methods and qualitative data analysis. | |
| *Relationship with participants* |  |  | |
| 6. Relationship established | Was a relationship established prior to study commencement? | Page 9, No prior personal or professional relationship with participants was reported. | |
| 7. Participant knowledge of the interviewer | What did the participants know about the researcher? e.g. personal goals, reasons for doing the research | Page 9. Before consent, participants were informed of the moderator’s role and the purpose of the study | |
| 8. Interviewer characteristics | What characteristics were reported about the inter viewer/facilitator? e.g. Bias, assumptions, reasons and interests in the research topic | Page 9-10 | |
| **Domain 2: study design** |  |  |  |
| *Theoretical framework* |  |  |  |
| 9. Methodological orientation and Theory | What methodological orientation was stated to underpin the study? e.g. grounded theory, discourse analysis, ethnography, phenomenology, content analysis | Page 10. An inductive–deductive analytic approach was used. Codes were consolidated into descriptive categories and subsequently mapped to the four predefined self-care support domains. The qualitative component was used to obtain focused stakeholder input for intervention development rather than to generate a standalone qualitative theory. |  |
| *Participant selection* |  |  |  |
| 10. Sampling | How were participants selected? e.g. purposive, convenience, consecutive, snowball | Page 8. Purposive sampling using predefined eligibility criteria. |  |
| 11. Method of approach | How were participants approached? e.g. face-to-face, telephone, mail, email | Page 8. Eligible participants were contacted directly by the principal researcher via WhatsApp. |  |
| 12. Sample size | How many participants were in the study? | Page 8. Two FGDs were conducted. The first included four patients with hypertension and four family caregivers; the second included five primary care nurses and three primary healthcare leaders (total n = 16). |  |
| 13. Non-participation | How many people refused to participate or dropped out? Reasons? | None; no invited participant declined participation or withdrew. |  |
| *Setting* |  |  |  |
| 14. Setting of data collection | Where was the data collected? e.g. home, clinic, workplace | Page 9. Both FGDs were conducted through Zoom. |  |
| 15. Presence of non-participants | Was anyone else present besides the participants and researchers? | Page 9. None; only the participants, moderator, and research assistant were present |  |
| 16. Description of sample | What are the important characteristics of the sample? e.g. demographic data, date | Pages 8–9; Supplementary Table 10 |  |
| *Data collection* |  |  |  |
| 17. Interview guide | Were questions, prompts, guides provided by the authors? Was it pilot tested? | Page 9 and Supplementary File 1. The stakeholder-specific interview guides were developed for this study and were not formally pilot-tested |  |
| 18. Repeat interviews | Were repeat inter views carried out? If yes, how many? | Page 9. No repeat FGDs were conducted. |  |
| 19. Audio/visual recording | Did the research use audio or visual recording to collect the data? | Page 9. Audio-video recordings were used. |  |
| 20. Field notes | Were ﬁeld notes made during and/or after the inter view or focus group? | Page 9. The research assistant maintained field notes. |  |
| 21. Duration | What was the duration of the inter views or focus group? | Page 9. Approximately 90 minutes for each FGD. |  |
| 22. Data saturation | Was data saturation discussed? | Page 9. Formal thematic saturation was not assessed and no claim of saturation was made. Sample adequacy was considered in relation to the focused study aim, participant specificity, depth of discussion, and complementary stakeholder perspectives. |  |
| 23. Transcripts returned | Were transcripts returned to participants for comment and/or correction? | Page 9. No. Transcripts were not returned to participants. Instead, the moderator conducted in-session clarification by summarizing key points and inviting participants to clarify or correct the researchers' interpretations during each FGD. |  |
| **Domain 3: analysis and ﬁndings** |  |  |  |
| *Data analysis* |  |  |  |
| 24. Number of data coders | How many data coders coded the data? | Pages 9–10. Two coders (NPARD and KANP) independently coded the transcripts. |  |
| 25. Description of the coding tree | Did authors provide a description of the coding tree? | Pages 9–10; Supplementary Tables 2–3. A formal coding tree was not produced. The process of code comparison, consolidation, and development of descriptive categories is reported in the Methods, and the evidence-to-module mapping is presented in Supplementary Tables 2–3. |  |
| 26. Derivation of themes | Were themes identiﬁed in advance or derived from the data? | Pages 9–10. An inductive–deductive approach was used. Codes and descriptive categories were derived from the data and subsequently mapped to the four predefined self-care support domains. Findings extending beyond those domains were retained for module refinement. |  |
| 27. Software | What software, if applicable, was used to manage the data? | Page 10. Data were managed and coded manually without qualitative data-analysis software. |  |
| 28. Participant checking | Did participants provide feedback on the ﬁndings? | Page 9. In-session clarification was undertaken during each FGD; no post-analysis participant checking was conducted. |  |
| *Reporting* |  |  |  |
| 29. Quotations presented | Were participant quotations presented to illustrate the themes/ﬁndings? Was each quotation identiﬁed? e.g. participant number | Pages 18 |  |
| 30. Data and ﬁndings consistent | Was there consistency between the data presented and the ﬁndings? | Yes. Pages 17–18 and Supplementary Tables 2–3. The reported stakeholder findings, illustrative quotations, and evidence-to-module mapping are presented consistently |  |
| 31. Clarity of major themes | Were major themes clearly presented in the ﬁndings? | Yes. Pages 16–17 and Supplementary Tables 2–3. The major descriptive categories and their translation into module components are reported. |  |
| 32. Clarity of minor themes | Is there a description of diverse cases or discussion of minor themes? | Pages 9–10 and 17–18; Supplementary Tables 2–3. Findings extending beyond the four predefined domains, including language clarity, family roles, cultural considerations, recording needs, nurse workflow, and follow-up mechanisms, were retained and reported. |  |
