# Supplementary material for: A dual-module person-centered nursing self-care bundle for hypertension management: development and expert validation
Source: BMC Nurs. 2026 Sep 26;25:987. doi: 10.1186/s12912-026-05377-2 (PMC13673632; doi:10.1186/s12912-026-05377-2)
Supplement: Supplementary file 5 [file 12912_2026_5377_MOESM5_ESM.docx]

**Supplementary File 5.** English Overview and Representative Excerpts of the Dual-Module Person-Centered Nursing-Based Self-Care Bundle

**Purpose**

This supplementary file provides an English-language overview and representative operational excerpts from the patient and nurse modules. The complete modules were developed in Bahasa Indonesia for adults aged 40–59 years with hypertension receiving primary care. The excerpts are provided to support scholarly appraisal and reproducibility without reproducing the complete copyrighted modules.

The patient module is intended as a patient-held self-care record and communication tool. The nurse module provides operational guidance for reviewing patient records, identifying barriers, providing person-centered feedback, documenting progress, and planning follow-up. The three-month sequence described below is the proposed protocol for subsequent feasibility and preliminary-effectiveness evaluation. If later testing supports implementation, patients are expected to continue completing the records daily, with review during routine monthly hypertension visits.

**1. Structure of the patient and nurse modules**

**Supplementary Table 5.1.** Overview of the patient module

| **Section** | **Main content** | **Operational function** |
| --- | --- | --- |
| 1. Introduction and module use | Purpose, intended users, instructions for patients, families, and healthcare professionals | Introduces daily recording and monthly review |
| 2. Understanding hypertension | Definition, risk factors, warning signs, and complications | Supports recognition of the condition and need for continued self-care |
| 3. Access to hypertension services | Primary care services, community programmes, financing, referral, and visit preparation | Helps patients prepare for and maintain contact with healthcare services |
| 4. Sociocultural and daily-life considerations | Dietary practices, cultural events, traditional practices, and feasible lifestyle adjustment | Supports culturally responsive self-care planning |
| 5. Family support | Medication and visit reminders, lifestyle support, emotional support, and shared caregiving roles | Clarifies optional and patient-preferred family involvement |
| 6. Four-domain self-care practice | Self-monitoring, lifestyle modification, medication regimen, and routine healthcare visits | Provides practical guidance, checklists, and recording forms |
| 7. Monthly feedback and follow-up | Monthly summary, barriers, questions, nurse feedback, agreed goals, and follow-up plan | Links home-based self-care records with clinical review |
| Appendices | Proposed implementation protocol and longitudinal monitoring forms | Supports standardized use during subsequent testing |

**Supplementary Table 5.2.** Overview of the nurse module

| **Section** | **Main content** | **Operational function** |
| --- | --- | --- |
| 1. Introduction and preparation | Purpose, target users, module preparation, and use of the patient-held record | Prepares nurses to implement the intervention |
| 2. Hypertension | Clinical overview, risk factors, warning signs, and complications | Supports accurate patient education and clinical review |
| 3. Person-Centered Nursing | PCN principles, communication, patient preferences, and shared decision-making | Guides person-centered interaction |
| 4. Access to hypertension services | Service pathways, community programmes, monitoring, and referral | Supports continuity and appropriate follow-up |
| 5. Cultural context | Cultural practices, dietary routines, treatment beliefs, and culturally responsive discussion | Helps nurses negotiate feasible recommendations |
| 6. Nursing role | Education, facilitation, monitoring, feedback, documentation, and family involvement | Defines operational nursing responsibilities |
| 7. Four-domain implementation | Self-monitoring assistance, lifestyle education, medication-regimen review, and routine-visit motivation | Structures education and monthly assessment |
| 8. Feedback and follow-up | Review procedures, scoring, individualized feedback, documentation, and maintenance planning | Converts patient records into follow-up actions |
| Appendices | Proposed implementation protocol, longitudinal scoring, and PCN–intervention mapping | Supports standardized use and subsequent evaluation |

**2. Operationalization of the four self-care domains**

**Supplementary Table 5.3.** Patient and nurse activities within each domain

| **Domain** | **Patient and family activities** | **Nurse activities** |
| --- | --- | --- |
| Self-monitoring assistance | Record blood-pressure readings when available; record symptoms and warning signs; note barriers to measurement | Review readings and symptoms; assess measurement barriers; clarify appropriate monitoring options; determine whether clinical review is needed |
| Lifestyle-modification education | Record dietary practices, physical activity, sleep, stress management, smoking, or alcohol-related difficulties | Explore routines and cultural context; identify one feasible lifestyle priority; provide individualized education and feedback |
| Medication-regimen monitoring | Record whether medication was taken as prescribed; document missed doses, availability problems, adverse effects, or concerns | Review adherence and barriers; explore beliefs without judgment; clarify concerns; refer medication-related decisions to the appropriate prescriber |
| Motivation for routine healthcare visits | Record scheduled visits, attendance barriers, questions, and topics for discussion | Review attendance and access barriers; respond to patient-selected concerns; agree on the next visit and follow-up actions |

**3. Proposed implementation protocol and intended routine use**

**Supplementary Table 5.4**. Proposed sequence of use

| **Time point** | **Patient and family activities** | **Nurse activities** |
| --- | --- | --- |
| Baseline | Receive the module; learn how to complete the records; identify an initial priority | Introduce the modules and recording procedures over approximately 10–15 minutes; demonstrate completion; agree on an initial patient-selected goal |
| Daily home use | Record self-care activities, medication use, blood-pressure readings when available, symptoms, barriers, and questions | No routine daily action required |
| Month 1 review | Bring the module and identify the most important concern for discussion | Review the first-month records; identify barriers; provide feedback; agree on a feasible goal; document the plan |
| Month 2 review | Continue daily recording and report progress or new difficulties | Assess consistency; review persistent or new barriers; adjust the goal and follow-up plan |
| Month 3 review | Bring the completed three-month records | Review trends, achievements, and unresolved barriers; agree on a maintenance plan |
| Intended routine use after successful evaluation | Continue daily recording | Review the record during routine monthly hypertension visits and revise the follow-up plan as required |

**Note:** The three-month sequence is a proposed pilot implementation protocol for subsequent evaluation. The duration of monthly review encounters has not yet been established and will be assessed during feasibility testing.

**4. Representative patient self-care record**

**Supplementary Table 5.5.** Example of a daily self-care record

| **Date** | **Blood pressure, if measured** | **Medication taken as prescribed** | **Lifestyle activity completed** | **Symptoms or warning signs** | **Barrier or difficulty** | **Question for the nurse** |
| --- | --- | --- | --- | --- | --- | --- |
| ____ | **/** mmHg | ☐ Yes  ☐ No | - Reduced salty food - Physical activity - Stress management | __________ | _________ | ______________ |

The form is repeated for daily use. Patients who do not have access to a home blood-pressure monitor may record measurements obtained from a primary healthcare center, community service, clinic, pharmacy, or another appropriate service.

**5. Representative monthly patient summary**

**Supplementary Table 5.6.** Monthly summary prepared for the routine visit

| **Monthly review item** | **Patient entry** |
| --- | --- |
| Number of days the record was completed | ______ days |
| Number of blood-pressure measurements | ______ |
| Self-care activity completed most consistently | ______________________________ |
| Activity that was most difficult | ______________________________ |
| Main symptom or concern | ______________________________ |
| Medication-related concern, if any | ______________________________ |
| Barrier to attending healthcare services | ______________________________ |
| Question or topic the patient wishes to discuss first | ______________________________ |
| Support requested from family or healthcare professionals | ______________________________ |

**6. Representative nurse review and documentation form**

**Supplementary Table 5.7.** Monthly nurse evaluation and individualized follow-up

Patient identifier: __________

Month: __________

Visit date: __________

Blood pressure at visit: / mmHg

Scoring:

0 = not undertaken

1 = rarely undertaken

2 = undertaken fairly regularly

3 = undertaken consistently according to the agreed plan

| **Domain** | **Score 0–3** | **Main patient-reported barrier** | **Nurse assessment and feedback** | **Agreed goal before the next visit** |
| --- | --- | --- | --- | --- |
| Self-monitoring | ☐0 ☐1 ☐2 ☐3 | _________________ | _________________ | _________________ |
| Lifestyle modification | ☐0 ☐1 ☐2 ☐3 | _________________ | _________________ | _________________ |
| Medication regimen | ☐0 ☐1 ☐2 ☐3 | _________________ | _________________ | _________________ |
| Routine healthcare visits | ☐0 ☐1 ☐2 ☐3 | _________________ | __________________ | __________________ |

Patient-selected priority:

Family-support role, if preferred by the patient:

Clinical review, referral, or additional support required:

☐ No

☐ Yes. Action: _______________________________________

Next review date: __________________

The scoring is used to support longitudinal discussion rather than to establish a clinical diagnosis. Patient records, reported barriers, preferences, symptoms, and clinical findings are considered together when determining follow-up actions. The modules already specify daily recording, optional family assistance, monthly review, and nurse documentation of feedback and realistic goals.

**7. Monthly review workflow**

At each monthly visit, the nurse:

1) confirms that the patient has brought the module and reviews the completeness of the records;
2) reviews blood-pressure readings, symptoms, medication use, lifestyle activities, and attendance at healthcare services;
3) asks the patient which concern or question should be discussed first;
4) identifies barriers within the four self-care domains;
5) checks for warning signs, medication concerns, persistently uncontrolled blood pressure, or other issues requiring clinical review;
6) provides focused education and individualized feedback;
7) agrees with the patient on one or two feasible priorities for the following month;
8) identifies the preferred role of family caregivers, where relevant;
9) documents the agreed actions, referral or additional support, and next review date.

Patient records are therefore not translated automatically into standardized advice. The nurse interprets the records together with the patient’s preferences, reported barriers, family context, and clinical condition before agreeing on individualized follow-up actions.

**8. Representative person-centered counseling prompts**

The nurse may use prompts such as:

1) “Which self-care activity was easiest for you to maintain?”
2) “Which activity was most difficult, and what made it difficult?”
3) “Which concern would you like us to discuss first today?”
4) “What change feels realistic for you before your next visit?”
5) “What support would be helpful from your family?”
6) “How can your family help without making you feel pressured?”
7) “Do you have concerns about your medication, access to medication, or possible adverse effects?”
8) “Did cultural, family, work, or social activities affect your plan this month?”
9) “What goal would you prefer to work on first?”

These prompts are illustrative and may be adapted to the patient’s needs, preferences, literacy, and clinical situation.

**9. Role of family caregivers**

Family involvement is optional and guided by patient preference. Family caregivers may:

1) remind the patient about medication and scheduled visits;
2) support healthy food choices and physical activity;
3) assist with reading or completing the record when required;
4) help the patient obtain a blood-pressure measurement;
5) accompany the patient to a routine visit;
6) provide emotional support without coercion, blame, or taking over the patient’s decisions.

The nurse assesses whether and how the patient wishes the family to be involved and documents an agreed, realistic family-support role. The underlying modules describe family assistance with reminders, checklist completion for patients who have reading or writing difficulties, and attendance at monthly visits when needed.

**10. Strategies for patients with limited health literacy**

The patient module uses:

1) plain-language explanations;
2) short instructions;
3) practical examples linked to daily routines;
4) check-box responses;
5) repeated summaries;
6) spaces for short written entries;
7) nurse demonstration during the baseline orientation;
8) verbal clarification during monthly visits;
9) optional assistance from a family caregiver;
10) prioritization of one or two feasible actions rather than multiple simultaneous changes.

When a patient has difficulty reading or writing, the nurse or a patient-approved family caregiver may assist with recording while ensuring that the responses reflect the patient’s own experiences and preferences.

**11. Availability of the complete modules**

The complete patient and nurse modules were developed in Bahasa Indonesia. They are available from the corresponding author upon reasonable request, subject to copyright and institutional requirements.
